# Effectiveness of non-pharmacological interventions in managing symptom clusters among lung cancer patients: a systematic review

**DOI:** 10.1186/s12885-024-13246-x

**Published:** 2024-12-06

**Authors:** Qiuhong Chen, Yonglin Li, Yiyang Lin, Xiujing Lin, Rachel Arbing, Wei-Ti Chen, Feifei Huang

**Affiliations:** 1 School of Nursing, Fujian Medical University, Fuzhou, Fujian China; 2 School of Nursing, University of California Los Angeles, Los Angeles, CA USA

**Keywords:** Lung cancer, Non-pharmacological intervention, Symptom cluster, Symptom management, Systematic review, Meta-analysis

## Abstract

**Background:**

Non-pharmacological interventions, as complements to pharmacological treatments, are widely employed for managing symptom clusters in patients with lung cancer. Although numerous systematic reviews and meta-analyses have explored the effects of these interventions, most studies have centred on the broader cancer population and specific symptom clusters. This review aims to consolidate existing non-pharmacological interventions and assess their effectiveness in managing symptom clusters among lung cancer patients.

**Methods:**

A comprehensive literature search, encompassing eight databases from inception to October 1, 2024, was conducted. Two independent reviewers carried out the study selection, quality assessment, and data extraction. Methodological quality was evaluated using the Cochrane Risk-of-Bias 2 tool and the Risk of Bias in Non-randomized Studies of Interventions. The findings were synthesized narratively based on intervention type and supplemented by meta-analysis using RevMan 5.4 software. The study protocol was registered with PROSPERO (CRD42023467406).

**Results:**

This systematic review comprised 15 relevant studies involving 1,692 patients, published between 2011 and 2024. The analysis revealed the effectiveness of psychological, educational, and complementary or alternative medicine interventions in alleviating the severity of most symptom clusters. However, the efficacy of exercise-based and multimodal interventions remained inconclusive. The meta-analysis demonstrated a positive impact of non-pharmacological interventions on depression compared with the control conditions (SMD = -0.30, 95% CI [-0.46, -0.15], *p* < 0.01, I^2^ = 6%). Additionally, the educational intervention subgroup showed low heterogeneity and effectively improved fatigue (SMD = -0.50, 95% CI [-0.68, -0.33], *p* < 0.01, I^2^ = 0%).

**Conclusions:**

Psychological and educational interventions have proven effective in managing symptom clusters in lung cancer patients. However, further research is needed to explore the effects of exercise, multimodal approaches, and complementary or alternative medicine. To enhance symptom management, future research could focus on core symptom clusters.

**Supplementary Information:**

The online version contains supplementary material available at 10.1186/s12885-024-13246-x.

## Introduction

Among all cancers, lung cancer has the highest mortality rate and the second-highest incidence rate, remaining a significant global health threat that places a burden on individuals and their families [1]. While advancements in treatment have led to improved survival rates [2], both lung cancer itself and its treatment can result in a range of unpleasant symptoms that are reported daily by lung cancer patients and health professionals [3]. These symptoms may occur simultaneously as a cluster, meaning that two or more interrelated, relatively stable symptoms co-occur [4, 5]. The symptoms experienced by lung cancer patients are diverse, with common symptom clusters including psychological, gastrointestinal, respiratory, and fatigue-related symptoms. As the disease and treatment progress, the severity, trajectory, and symptom composition of each symptom cluster undergo dynamic changes [6]. An eight-year prospective cohort study by Cheville et al. [7] showed that fatigue, dyspnoea and cough were persistent symptom clusters up to 5 years after the diagnosis of lung cancer.

While there is currently no unified consensus on the specific symptom cluster experienced by lung cancer patients, empirical evidence has shown that the synergistic effect of symptoms in the cluster leads to a greater negative impact on patients than a single symptom [8]. These symptom clusters can result in adverse side effects, including the interruption of functional ability, impaired role and social relationships, and the exacerbation of underlying illnesses [9]. These factors ultimately lead to a decrease in quality of life (QOL) and a worsened prognosis [10, 11]. Therefore, the effective management of symptom clusters among lung cancer patients is a priority in oncology. Compared with drug therapy, non-pharmacological treatment has the advantages of high safety, large economic benefits and long-term availability. In addition, non-pharmacological intervention programs can be designed on the basis of patient preferences and abilities, making them more acceptable [12]. As a result, an increasing number of non-pharmacological interventions, complementing pharmacological approaches, have been employed to manage symptom clusters in patients with lung cancer [13, 14].

However, there is some disagreement as to whether non-pharmacological interventions are effective for treating symptom clusters in lung cancer patients. While some original studies [15–17] reported a positive impact of non-pharmacological interventions on reducing the severity of symptom clusters in lung cancer patients, Chen et al. [18], Cheung et al. [19], and Molassiotis et al. [20] found no significant differences between the intervention and control groups. In addition, the diversity of non-pharmacological interventions makes it difficult to determine the efficacy of a certain element. Therefore, it is important to assess the value of non-pharmacological interventions in the management of lung cancer symptom clusters using an evidence-based approach. This can not only provide a comprehensive and systematic evidence base and reduce bias and error but also guide the formulation and application of non-pharmacological intervention programs for lung cancer patients, to alleviate their symptom burden and improve their symptom management ability.

Currently, systematic reviews predominantly focus on cancer populations in general, with particular emphasis on specific breast cancer populations, and are primarily concerned with evaluating specific symptom clusters. For instance, So et al. [21] categorized non-pharmacological interventions into body-based, cognitive-behavioural, and educational interventions. These factors were observed to reduce the severity of symptom clusters and enhance the QOL and functional ability of cancer patients. A meta-analysis of 10 studies provided preliminary evidence supporting the benefits of qigong interventions for sleep disturbance-related symptom clusters in cancer patients [22]. Wong et al. [23], in their study encompassing 16 studies, concluded that various non-pharmacological interventions were effective in treating the fatigue-sleep disturbance-depression symptom cluster in breast cancer patients undergoing chemotherapy.

In terms of lung cancer-related studies, only one systematic review was found. Yorke et al. [24] assessed the effectiveness of non-pharmacological interventions in alleviating respiratory symptoms, such as breathlessness, cough, and haemoptysis, in lung cancer patients. However, this review focused solely on interventions for one symptom cluster and was published a decade ago, excluding more recent studies, particularly those from Asia. Currently, no systematic review has provided a comprehensive overview of the effectiveness of non-pharmacological interventions in managing symptom clusters in patients with lung cancer.

Therefore, this study aimed to review currently available non-pharmacological interventions and assess their effectiveness in managing symptom clusters in lung cancer patients. We addressed the following questions: (1) What are the current non-pharmacological interventions for lung cancer symptom clusters? (2) What is the effectiveness of non-pharmacological interventions in managing symptom clusters and single symptoms in lung cancer patients?

## Methods

The protocol for this review was registered with PROSPERO under reference number [CRD42023467406]. It adheres to the Preferred Reporting Items for Systematic Reviews and Meta‐Analyses (PRISMA) statement [25].

### Eligibility criteria

The PIPOST model [26] was used, as follows.

#### P (Participants)

Inclusion criteria: Patients with lung cancer, aged 18 years or above.

#### I (Intervention)

### Inclusion criterion

Non-pharmacological interventions such as psychoeducational, exercise, and cognitive-behavioural interventions.

### Exclusion criterion

Studies involving pharmacological interventions, either implemented alone or in combination with non-pharmacological interventions.

#### P (Professional)

### Inclusion criteria

Nursing staff, physicians, therapists, or other health care provider.

#### O (Outcome)

### Inclusion criteria

Primary outcomes were symptom clusters identified by occurrence, frequency, intensity, or distress, and measured by multi-dimensional symptom or individual symptom questionnaires. The secondary outcomes included QOL, functional ability, physical performance, one-year survival, length of hospital stay, mood state, activity levels, circadian rhythms, global health status, anxiety and depression, and cancer symptoms (pain, fatigue, nausea, sleep disturbance, distress, shortness of breath, difficulty remembering, lack of appetite, drowsiness, dry mouth, sadness, vomiting, and numbness).

#### S (Setting)

### Inclusion criteria

Hospitals (including wards and outpatient clinics), specialist cancer centres, mental health clinics, communities, or families.

#### T (Type of evidence)

### Inclusion criteria

Randomized controlled trials (RCTs) or quasi-experimental trials.

### Exclusion criteria

Conference abstracts, reviews, editorials, dissertations, letters, books, unpublished manuscripts, etc.; duplicate studies: for studies published with the same or different titles, or in more than one journal, the most updated version was considered; studies that had insufficient data or were unavailable in full text after contacting the original authors; or studies reported in languages other than English or Chinese.

### Search strategy

A comprehensive literature search was conducted in October 2024. Eight databases were utilized in this search: PubMed, Web of Science, EMBASE, CINAHL, Cochrane Library, China National Knowledge Infrastructure, Wanfang database, and VIP Database for Chinese Technical Periodicals. We considered studies published from the inception of the database to October 1, 2024. To ensure that no relevant literature was overlooked, a supplementary search was also carried out on Google Scholar, and a snowball search was performed by screening the reference lists of all pertinent studies. Appendix 1 presents the search strategy for PubMed.

### Study selection

After removing duplicate articles using the reference management software Endnote 20, two researchers independently screened titles and abstracts to determine the inclusion or exclusion of these studies on the basis of established criteria. The full texts of these potentially eligible studies were then retrieved and reassessed by the researchers. Any disagreement between the two reviewing researchers regarding study eligibility was resolved through discussion with a senior researcher. The overall weighted kappa coefficient was 0.743 (*p* < 0.05).

### Quality assessment

After two rounds of screening, two independent researchers assessed the quality of the identified studies. The Cochrane Risk-of-Bias 2 (RoB 2) tool for randomized trials and the Risk-of-Bias in Non-randomized Studies of Interventions (ROBINS-I) tool for quasi-experimental trials were utilized. RoB 2 evaluates bias risk in five domains: randomization, intervention adherence, outcome data completeness, outcome measurement, and result reporting [27]. In each domain, reviewers classified bias as “low risk”, “high risk”, or “some concerns”. The ROBINS-I assesses bias across seven domains: confounding, participant selection, intervention classification, adherence to interventions, missing data, outcome measurement, and result reporting [28]. Reviewers assigned bias levels as “low risk”, “moderate risk”, “serious risk”, or “critical risk” in each domain. Any disagreements were resolved through discussion or with the assistance of a senior researcher. The overall weighted kappa coefficient was 0.755 (*p* < 0.05). See Appendices 2–3 for findings related to the study quality assessments.

### Data extraction and summary

Two independent researchers extracted the following data from the original articles: author, year of publication, country, study design, study setting, participant characteristics, symptom clusters, intervention characteristics, control group details, outcome measures, measurement timepoints, findings within groups, and information on adverse events. Any discrepancies were addressed by consulting the original literature or through discussion with a senior researcher.

### Data analysis

The findings were categorized based on types of interventions and reported outcomes and then summarized and synthesized into a narrative format. In synthesizing data across studies, effect sizes were standardized and expressed as Cohen's d, which quantifies the difference in means between the intervention and control groups. We followed Cohen's conventions [29] for interpreting effect sizes: 0 < Cohen's d ≤ 0.2 indicated a small effect, 0.2 < Cohen's d ≤ 0.8 indicated a medium effect, and Cohen's d > 0.8 indicated a large effect.

We conducted a meta-analysis to evaluate the effects of non-pharmacological interventions on individual symptoms using Review Manager (RevMan) software (version 5.4). Given the high heterogeneity in the measurement tools among the included studies, we employed the standardized mean difference (SMD) and 95% confidence interval (CI) to aggregate the results. Statistical significance was considered at *p* < 0.05. The I^2^ test was utilized to assess heterogeneity [30]. If* p* > 0.1 and I^2^ < 50%, the included studies were deemed homogeneous and the pooled results were analyzed using a fixed-effects model. Conversely, if I^2^ > 50%, a high degree of heterogeneity between studies was indicated which necessitated an exploration of its potential sources through sensitivity and subgroup analyses. In this study, subgroup analyses were grouped according to intervention type. Since the time points of measurement varied across studies, we retained the final measurement for the meta-analysis of studies that included multiple time points. In addition, to assess publication bias, we mapped funnel plots for the outcomes of 10 or more included studies by RevMan software and performed Egger's regression tests using Stata software (version 17.0), with *p*-values < 0.05 considered statistically significant publication bias.

## Results

### Search results

**Fig. 1:**
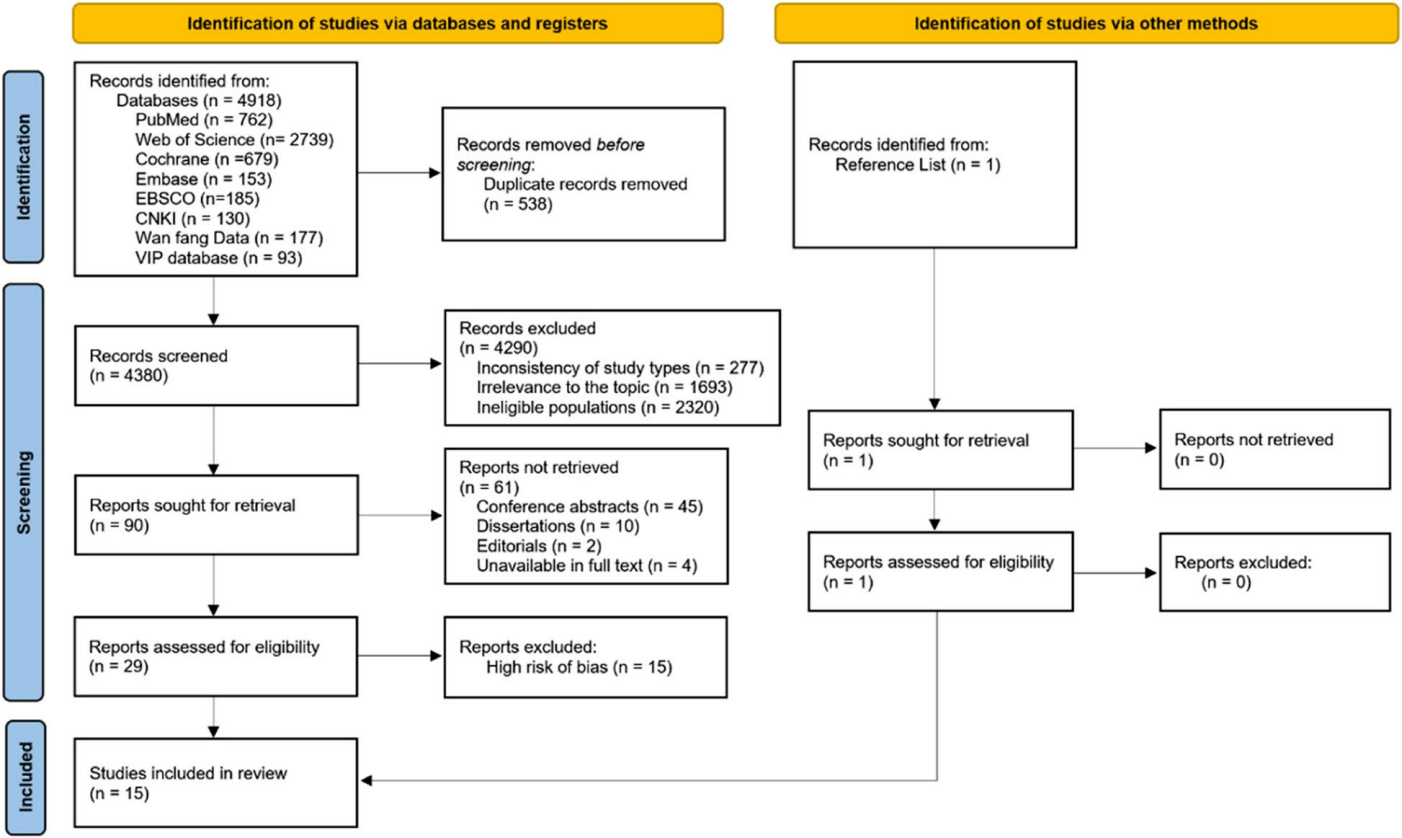
Flow diagram of the study selection

A total of 4,918 citations were identified in the initial search. After removing duplicates, 4,380 studies remained. Following the screening of titles and abstracts, 4,290 studies were excluded due to inconsistency in study types, irrelevance to the topic, or ineligible populations. Upon reviewing the full texts of the remaining 90 studies, 61 were subsequently removed. One study was supplemented by a snowballing search. The quality of the remaining 30 studies was assessed, leading to the exclusion of 15 studies that were rated as having a high risk of bias. Ultimately, 15 studies were included, comprising 7 RCTs rated as low risk for bias and 6 RCTs rated as some concern of bias, and 2 quasi-experimental studies rated as low risk or moderate risk of bias. The PRISMA flow chart illustrating this process is shown in Fig. 1.

### Study characteristics

The 15 studies included in this review were published between 2011 and 2024, with 60% (*n* = 9) appearing in the last five years (2020 to 2024). The majority of studies employed an RCT design (*n* = 13, 87%). Eight studies were conducted in mainland China, two in the Hong Kong Special Administrative Region of China, two in England, and the remaining three in Vietnam, Thailand, and Taiwan, China. Five of the studies were conducted in outpatient settings, including radiotherapy outpatient clinics, oncology and respiratory medicine outpatient clinics, as well as psychological nursing clinics. Twelve were single-centre studies and the remaining three were multi-site studies.

Furthermore, the 15 included studies encompassed a total of 1,692 participants, with sample sizes ranging from 30 to 263. The average age of the participants varied, ranging from 45.15 (SD = 4.34) to 69.5 (SD = 10.20) years. The majority of participants had advanced-stage lung cancer (stage III or IV). In terms of treatment methods, chemotherapy was the most commonly adopted approach, followed by surgery and radiotherapy. Further details about the characteristics of the included studies can be found in Table 1.

**Table 1:** Characteristics of the included studies

No	Author, Year; Country	Study design	Study setting	Participant characteristic					Symptom cluster	Intervention	Control	Bias
				**Sample size total (IG:CG)**	**Diagnosis stage** **(%)**	**Mean age** **(SD)**	**Gender** **(%)**	**Current treatment**				
1	Chan et al., 2011 [31]; Hong Kong, China	RCT	Outpatient radiotherapy unit of a public hospital	140 (70:70)	Unspecified	Unspecified	Male (83%) Female (17%)	Undergoing radiotherapy	Breathlessness, fatigue, anxiety	Psychoeducational intervention	Usual care	Low
2	Chen et al., 2015 [18]; Taiwan, China	RCT	Medical centre	116 (58:58)	Stage I (64.7%) Stage II (7.8%) Stage III (9.5%) Stage IV (7.8%)	64.16 (10.89)	Male (46.6%) Female (53.4%)	No treatment (31.0%) Operation (54.3%) Chemotherapy (0.9%) Radiotherapy (3.4%) Target therapy (7.8%) Chemotherapy and radiotherapy (2.6%)	Pain, fatigue, nausea, sleep disturbance, sadness, shortness of breath, difficulty remembering, poor appetite, drowsiness, dry mouth, distress, vomiting, numbness	Home-based walking exercise	Usual care	Low
3	Cheung et al., 2021 [19]; Hong Kong, China	RCT	Oncology and respiratory medicine out-patient clinics of a public hospital	30 (10:9:11) (IG1:IG2:CG)	Unspecified	Aerobic exercise group: 61.00 (12.12) Tai-chi group: 61.11 (7.01) Control group: 58.36 (9.32)	Male (53.3%) Female (46.7%)	Targeted therapy (60.0%) Non-targeted therapy (40%) Chemotherapy (23.3%) Radiotherapy (3.3%) No treatment (13.3%)	Sleep disturbance, anxiety, depression, fatigue	IG1: Aerobic exercise intervention IG2: Tai-chi intervention	Self-management	Low
4	Khamboon et al., 2021 [15]; Thailand	Quasi‑experimental study	University hospital in the lower northern region of Thailand	80 (40:40)	Stage III (5.0%) Stage IV (95.0%)	IG: 61.58(6.28) CG: 60.43(6.13)	Male (57.5%) Female (42.5%)	Undergoing chemotherapy	Fatigue, loss of appetite, anxiety	Symptom cluster management intervention based on symptom management theory	Usual care	Low
5	Molassiotis et al., 2021 [20]; Vietnam	RCT	National Lung Hospital and Nam Dinh General Hospital	156 (78:78)	Stage I (2.6%) Stage II (6.4%) Stage III (29.5%) Stage IV (61.5%)	56.84 (9.45)	Male (74.4%) Female (25.6%)	Chemotherapy (41.0%) Chemotherapy and radiotherapy (38.5%) Chemotherapy and operation (20.5%)	Breathlessness, fatigue, anxiety	Qigong training	Usual care	Low
6	Yorke et al., 2022 [32]; England	RCT	8 hospitals	263 (132:131)	Unspecified	IG: 69.2 (8.70) CG: 69.5 (10.20)	Male (49.8%) Female (50.2%)	Unspecified	Breathlessness, cough, fatigue	Respiratory Distress Symptom Intervention (RDSI)	Usual care	Low
7	Yorke et al., 2015 [33]; England	RCT	11 participating centres: 7 secondary care teaching hospitals, 2 specialist cancer centres and 2 district general hospitals	101 (50:51)	Unspecified	IG: 67.8 (10.1) CG: 67.6 (9.1)	Male (46.5%) Female (53.5%)	Unspecified	Breathlessness, cough, fatigue	Respiratory Distress Symptom Intervention (RDSI)	Usual care	Some concern
8	Jiang et al. [34], 2022; China	RCT	Chest hospital	108 (59:59)	Stage I (78.0%) Stage II/III (22.0%)	Unspecified	Male (33.9%) Female (66.1%)	Undergoing surgery	- Cough, expectoration, haemoptysis, chest tightness, shortness of breath - Pain, fatigue, disturbed sleep, drowsiness - Distress, sadness - Nausea, vomiting, lack of appetite, weight loss, constipation - Dry mouth, difficulty remembering, numbness	Auricular acupressure therapy	Usual care	Some concern
9	Li et al., 2017 [35]; China	RCT	Cardiothoracic surgery in a general hospital	57 (28:29)	Unspecified	Unspecified	Male (61.4%) Female (38.6%)	Undergoing surgery	Pain, insomnia, fatigue	Peri-operative cognitive-behavioural intervention	Usual care	Moderate
10	Li et al., 2018 [16]; China	RCT	Psychological nursing clinic in a general hospital	120 (60:60)	Unspecified	Unspecified	Male (53.3%) Female (46.7%)	Unspecified	Pain, insomnia, fatigue	Reception-assessment-narrative care-individualised psychological intervention- outpatient follow up combined with telephone follow up	Reception- assessment- personalized psychological intervention -telephone follow-up	Low
11	Lu et al., 2022 [36]; China	RCT	Department of respiratory and critical care medicine in a general hospital	57 (28:29)	Stage I (12.3%) Stage II (36.8%) Stage III (38.6%) Stage IV (12.3%)	IG: 54. 93 (11.59) CG: 55.62 (10.81)	Male (68.4%) Female (31.6%)	Undergoing chemotherapy	Pain, fatigue, nausea, sleep disturbance, sadness, shortness of breath, difficulty remembering, poor appetite, drowsiness, dry mouth, distress, vomiting, numbness, cough, constipation, sore throat	Professional integration management	Usual care	Some concern
12	Wei et al., 2020 [17]; China	RCT	Department of Medical Oncology in a general hospital	108 (54:54)	Stage I (7.4%) Stage II (11.1%) Stage III (55.6%) Stage IV (25.9%)	IG: 57.15 (8.84) CG: 56.94 (7.62)	Male (56.5%) Female (43.5%)	Undergoing chemotherapy	Fatigue, pain, sleep disturbance	Precision care based on multidisciplinary collaborative model	Usual care	Some concern
13	Yang et al., 2020 [37]; China	RCT	Outpatient clinic in a general hospital	144 (74:70)	Stage III (68.8%) Stage IV (31.3%)	Unspecified	Male (46.5%) Female (53.5%)	Taking oral chemotherapy drugs	Pain, fatigue, nausea, sleep disturbance, distress, shortness of breath, difficulty remembering, lack of appetite, drowsiness, dry mouth, sadness, vomiting, numbness	Multinational Association for Supportive Care in Cancer Oral Agent Teaching Tool (MOATT) based intervention	Routine outpatient intervention	Some concern
14	Ying et al., 2019 [38]; China	RCT	A general hospital	82 (41:41)	Stage II (20.7%) Stage III (36.6%) Stage IV (42.7%)	IG: 45.15 (4.34) CG: 45.24 (4.39)	Male (61.0%) Female (39.0%)	Undergoing chemotherapy	Fatigue, pain, sleep disturbance	Symptom cluster management program intervention	Usual care	Some concern
15	Zhang et al., 2024 [39]; China	RCT	Department of Oncology in a general hospital	130 (65:65)	Stage I–II	IG: 58.85 (10.80) CG: 58.46 (11.60)	Male (53.8%) Female (46.2%)	Chemotherapy (73.1%) Radiotherapy (10.8%) Combination therapy (16.2%)	Cough, expectoration, shortness of breath	Acupuncture	Usual care	Low

*Note: IG* intervention group, *CG* control group, *SD* standard deviation

### Intervention characteristics

Nine studies employed individual interventions, two utilized group interventions, and the remaining four employed a combination of both individual and group approaches. With respect to interventionists, eight studies (53%) exclusively utilized nurses, whereas the other studies involved professional athletic coaches or multidisciplinary teams. The minimum duration of intervention was one-week, whereas the maximum duration spanned six-months. The frequency of interventions ranged from two to 36 sessions. Six studies followed patients for varying durations, ranging from 3 weeks to 9 months after the intervention. All studies employed face-to-face interventions, with approximately 86% (*n* = 12) incorporating telephone follow-ups and 29% (*n* = 4) utilizing the WeChat platform for communication. Five studies designed interventions based on established theories, models, or frameworks. For instance, Khamboon and Pakanta [15], and Li et al. [35] developed cognitive-behavioural intervention programs utilizing Symptom Management Theory and Ellis's “ABC” theory, respectively. Chan et al. [31] devised an analytical model to elucidate the connection between psychoeducational interventions and their outcomes. Yorke et al. [32, 33] applied the Medical Research Council framework to create and assess complex interventions. The full intervention characteristics of the studies included in this review are detailed in Table 2.

**Table 2:** Intervention characteristics of the included studies

No	Author, Year	Intervention type	Theoretical framework	Sessions	Timing (duration)	Delivery format	Interventionist	Activity (content)	Participants	Medium	Measurement timepoints	Adverse events
1	Chan et al., 2011 [31]; Hong Kong, China	Educational intervention	NR	4 sessions (40 min per session)	1 week before and 3 weeks after RT, once a week	Individual	RNs with two years of clinical experience	- 40 min educational package plus coaching of PMR was delivered within 1 wk prior to beginning course of RT and 3 wks after commencing RT - Encouraged to practice PMR daily and as required	70 lung cancer patients undergoing RT	Face-to-face intervention, telephone follow-up	- Baseline - End of intervention - Follow-up after 3 weeks - Follow-up after 9 weeks	None
2	Chen et al., 2015 [18]; Taiwan, China	Exercise intervention	NR	36 sessions (40 min per session)	12 weeks, 3 times a week	Individual	Researchers	- Walking exercise booklet used to instruct on mode, intensity and frequency of exercise; pulse-rate measurement; Borg’s rating of perceived exertion scale; prevention of sports injuries; and termination time point of exercise - Instructed to achieve a target heart rate of 60–80% - Weekly telephone interviews to discuss exercise experiences and to complete exercise logs	58 lung cancer patients	Face-to-face intervention, telephone follow-up	- Baseline - End of intervention - Follow-up after 3 months	None
3	Cheung et al., 2021 [19]; Hong Kong, China	Exercise intervention	NR	24 sessions (60 min per session)	12 weeks, twice a week	Group	Aerobic exercise IG: 2 licensed exercise trainers Tai-chi IG: Tai-chi master > 5 yrs of teaching experience	**Aerobic exercise IG:** - Both aerobic and strengthening exercises in each class (30 min each) - Aerobic exercises included walking on a treadmill and cycling on a stationary bike - Increase arm, leg and abdominal strength and improve trunk stability through strengthening exercises - Instructed to perform moderate-intensity aerobic exercise for at least 90 min/wk during intervention period. After intervention, practice moderate-intensity aerobic exercise for at least 150 min/wk and practice 2 sets of strengthening exercises with 10 reps each on alternate days - Standardized exercise log used **Tai-chi IG:** - Class based on 24-form Yang style of tai-chi exercise set. Each session included warm-up, guided run-through of movements, breathing techniques, relaxation in tai-chi, and cooldown. Tai-chi theory and principles of techniques explained - During intervention period, encouraged to practice tai-chi (30 min) at least 3 x/wk. After intervention, encouraged to practice at least 5 x/wk (150 min total) - Standardized exercise log used	19 lung cancer patients (Aerobic exercise: Tai-chi = 10:9)	Face-to-face intervention	- Baseline - End of intervention - Follow-up after 3 months - Follow-up after 9 months	None
4	Khamboon et al., 2021 [15]; Thailand	Psychological intervention	SMT	4 sessions (5—45 min per session)	28 days total, specifically on day 1, 7, 14, and 28	Individual	Specially trained nurse	- Session 1: inaccuracy and misconceptions about symptom clusters clarified and discussed - Session 2: evidence-based guidance provided on physical, behavioural and psychological strategies including exercise guidance, dietary guidance, relaxation and distraction techniques to manage symptom clusters - Session 3: at home telephone call to check‑in, identify concerns, and provide emotional support - Session 4: severity of symptom clusters evaluated systematically	40 lung cancer patients undergoing chemotherapy	Outpatient face-to-face intervention, telephone follow-up	- Baseline - Day 7 - Day 14 - End of intervention	None
5	Molassiotis et al., 2021 [20]; Vietnam	Exercise intervention	NR	4 sessions (90 min per session)	6 weeks (2 weeks intervention and 4 weeks of self-directed practice)	Group	Professional Qigong coach with 12 yrs of experience	- Qigong training involved series of simple, repeated practices including body posture/movement, breathing practice, and meditation performed in synchrony - IG received a 90 min Qigong training, 2 x/wk for first 2 wks. In next 4 wks, asked to practice Qigong at home for at least 30 min/day, 5 days/wk - After 6 wk intervention period, started additional 6 wk unsupervised practice with log of daily activity during follow-up period	78 lung cancer patients	Face-to-face intervention, telephone follow-up	- Baseline - End of intervention - Follow-up after 6 weeks	None
6	Yorke et al., 2022 [32]; England	Multimodal intervention	NR	2 sessions (1 face-to-face group session and 1 follow-up refresher session, 180 min per session)	4 weeks	Individual	Healthcare professionals, including nurses, PTs and OTs	- Controlled breathing techniques: diaphragmatic breathing exercises or pursed lip breathing, practiced 2 x/day or used as needed for episodes of intense breathlessness and/or anxiety - Cough suppression techniques: education (capacity for voluntary cough easing), identify warning signs of cough and replace with sips of water, modified swallow technique, huff cough technique or relaxed throat breathing - Acupressure: 6 acupressure points taught: L7, L9 (for cough and dyspnoea, located on wrist areas), LI4 (for energy, located in hand), CV21 and CV22 (for cough and dyspnoea, located in sternum) and ST36 (for energy, located in knee). Could select any points in any combination to apply pressure for 1 min at least 2 x/day for symptom relief - Exercise: Individually tailored exercise plan, e.g., walking incrementally increasing distances and incorporating breathing techniques as required	132 lung cancer patients	Face-to-face intervention, telephone follow-up	- Baseline - End of intervention - Follow-up after 8 weeks	None
7	Yorke et al., 2015 [33]; England	Multimodal intervention	NR	2 sessions (180 min per session)	4 weeks	Individual	Specialist nurses, PTs and complementary therapists	Intervention program same as Yorke et al. (2022)	50 lung cancer patients	Face-to-face intervention, telephone follow-up	- Baseline - End of intervention - Follow-up after 8 weeks	None
8	Jiang et al. [34], 2022; China	CAM intervention	NR	NR	From 1 day before surgery to 6 days after surgery (1 week)	Individual	Researchers	- After confirming the integrity of the auricle skin 1 day before surgery, 10 acupoints selected and magnetic pellets secured to each acupoint - Asked to press pellets 5 x /day (i.e., morning, 30 min before breakfast, lunch and dinner, and before bedtime) for 30 s each acupoint	59 lung cancer patients undergoing surgery	Face-to-face intervention	- Baseline - Day 1 after surgery - Day 3 after surgery - End of intervention	None
9	Li et al., 2017 [35]; China	Psychological intervention	Ellis's “ABC” theory	8 sessions (30—40 min per session)	From 1st day after admission to 2nd day before discharge	Individual	Researchers	**Rational-emotive therapy face-to-face talks:** - Session 1: Understand emotional and symptomatic experience; irrational beliefs leading to emotional and symptom experiences preliminarily analysed; introduced to ABC theory - Session 2: Symptom experience and cognitive bias of patients collected to guide understanding of irrational beliefs vs own symptoms; identify and define problems positively and find reasonable solutions - Session 3: Cognitive behavioural techniques used to facilitate giving up irrational beliefs and relieving uncomfortable symptoms - Session 4: Feedback and consolidate intervention effect **Relaxation training course:** - Session 1: Instructed to master key points of PMR and abdominal breathing. Patients were instructed by the researchers to complete the 1st practice - Session 2—4: Interventionists demonstrated relaxation techniques and guided 1 exercise (about 20 min)	28 lung cancer patients undergoing surgery	Face-to-face intervention	- Baseline - 3 days after surgery - End of intervention	None
10	Li et al., 2018 [16]; China	Psychological intervention	NR	4 sessions (45—60 min per session)	4 weeks, once a week	Individual	Nurse-led outpatient mental care staff (13 in total)	- Choose preferred narrative story and record high-frequency words, emotional keywords, coping methods and feelings of patients in the narrative process - After patient narration, positive feedback provided, key links reviewed, and assisted to find own strengths, achievements and efforts in story - Helped patients to externalize the problem and guided patients to build self-confidence - Instructed to perform relaxation training - Homework given	60 advanced lung cancer patients	Outpatient face-to-face intervention, telephone follow-up	- Baseline - End of intervention	None
11	Lu et al., 2022 [36]; China	Multimodal intervention	NR	NR	6 months	Individual and group	Multidisciplinary team included specialist nurses of nutrition, pain and physical and mental medicine depts, and head nurse of respiratory and critical care medicine dept was team leader	- Individual guidance: content includes nutrition, activity, respiratory muscle training, and relaxation training - WeChat platform interaction: weekly online health education provided through WeChat communication platform—how to monitor body weight, regulate diet, exercise respiratory muscles, control pain, promote sleep, etc - Long-term behaviour promotion: record daily task completion and changes in symptoms, feedback on existing or potential problems; encouraged to connect with WeChat every week	28 lung cancer patients undergoing chemotherapy	Telephone follow-up, WeChat platform communication, family visits, special lectures, and patient networking activities	- Baseline - End of intervention	None
12	Wei et al., 2020 [17]; China	Multimodal intervention	NR	NR	Enrolment to 1 month after discharge	Individual and group	Multidisciplinary group: 1 head nurse and 1 attending physician in medical dept of oncology, 1 psychological consultant, 1 dietitian, 1 pain, tumor and nutrition specialist nurse, and 1 nursing graduate student	- Assessments: nutritional status, fatigue degree, pain intensity and insomnia, causes and duration of fatigue/cancer pain/sleep, and psychological status - Information sharing: assessment results sent to WeChat group - Focused discussion used to develop individualised precision nursing plans - Promote collaboration: if symptoms change during chemotherapy, ask other clinicians or specialist nurses to assist - Follow-up: After discharge, followed up by telephone 2 x/wk	54 lung cancer patients undergoing chemotherapy	Face-to-face intervention, telephone follow-up, WeChat group	- Baseline - End of intervention	None
13	Yang et al., 2020 [37]; China	Educational intervention	NR	NR	6 months	Individual and group	Outpatient physicians and nurses	- Evaluation phase: critical issue assessment - General education stage: general education before taking medication - Special medicine education stage: special information involved in taking medications provided - Evaluation phase: evaluated mastery	74 lung cancer patients with oral chemotherapy drugs	Face-to-face intervention, telephone follow-up, WeChat group	- Baseline - End of intervention	None
14	Ying et al., 2019 [38]; China	Multimodal intervention	NR	NR	3 weeks	Individual and group	Researchers	- Symptom knowledge: causes of pain, fatigue and sleep disorders explained, corrected wrong cognition of patients, standardized use of analgesics, and delivered sleep hygiene education - Nutrition exercise intervention: nutritional intervention carried out according to dietary principles. Instructed to perform moderate-intensity exercise 5 x/wk - Skill approach intervention: breathing exercises, relaxation and imagery training, and stimulus control. Instructed to fill out symptom management diary	41 lung cancer patients undergoing chemotherapy	Face-to-face intervention, telephone follow-up, WeChat group communication	- Baseline - End of intervention	None
15	Zhang et al., 2024 [39]; China	CAM intervention	NR	NR	7 days	Individual	Traditional Chinese medicine specialist nurses	- Acupuncture needles were inserted at a 90° angle into the skin at the following bilateral acupoints: LU7 (Lie Que), LU9 (Tai Yuan), BL13 (Fei Shu), and BL20 (Pi Shu) - The acupuncture was maintained for 3 days by leaving the intradermal needles inserted for 3 days. After a 1-day interval, the needles were re-inserted and left in place for 3 days - During the retention period, the patients were told to apply pressure 5–10 times per day for 1 min per point and at a depth of 0.5–1 cm, with sufficient strength to cause the points to be slightly swollen and painful	65 lung cancer patients	Face-to-face intervention	- Baseline - End of intervention	Within the intervention group, two patients were allergic to the adhesive tape

*Note: NR* not reported, *PMR* progressive muscle relaxation, *RT* radiotherapy, *SMT* Symptom Management Theory, *IG* intervention group, *CAM* complementary or alternative medicine, *PT* physical therapist, *OT* occupational therapist

We classified the interventions in the study into five groups: a) Educational interventions—encompassed psychoeducational interventions and medication health education based on the Multinational Association for Supportive Care in Cancer Oral Agent Teaching Tool (MOATT) [37]; b) Exercise interventions—home-based walking, aerobic exercise, tai chi, and qigong training. c) Psychological interventions—cognitive-behavioural interventions and narrative care; d) Multimodal interventions—combine two or more approaches, as exemplified by the respiratory distress symptoms intervention programme developed by Yorke et al. [33], which incorporates breath control, cough suppression, acupressure, and exercise; and e) Complementary or alternative medicine (CAM) interventions—specifically involving auricular acupressure therapy and acupuncture.

### Effectiveness of the intervention on symptom cluster/outcome

**Fig. 2:**
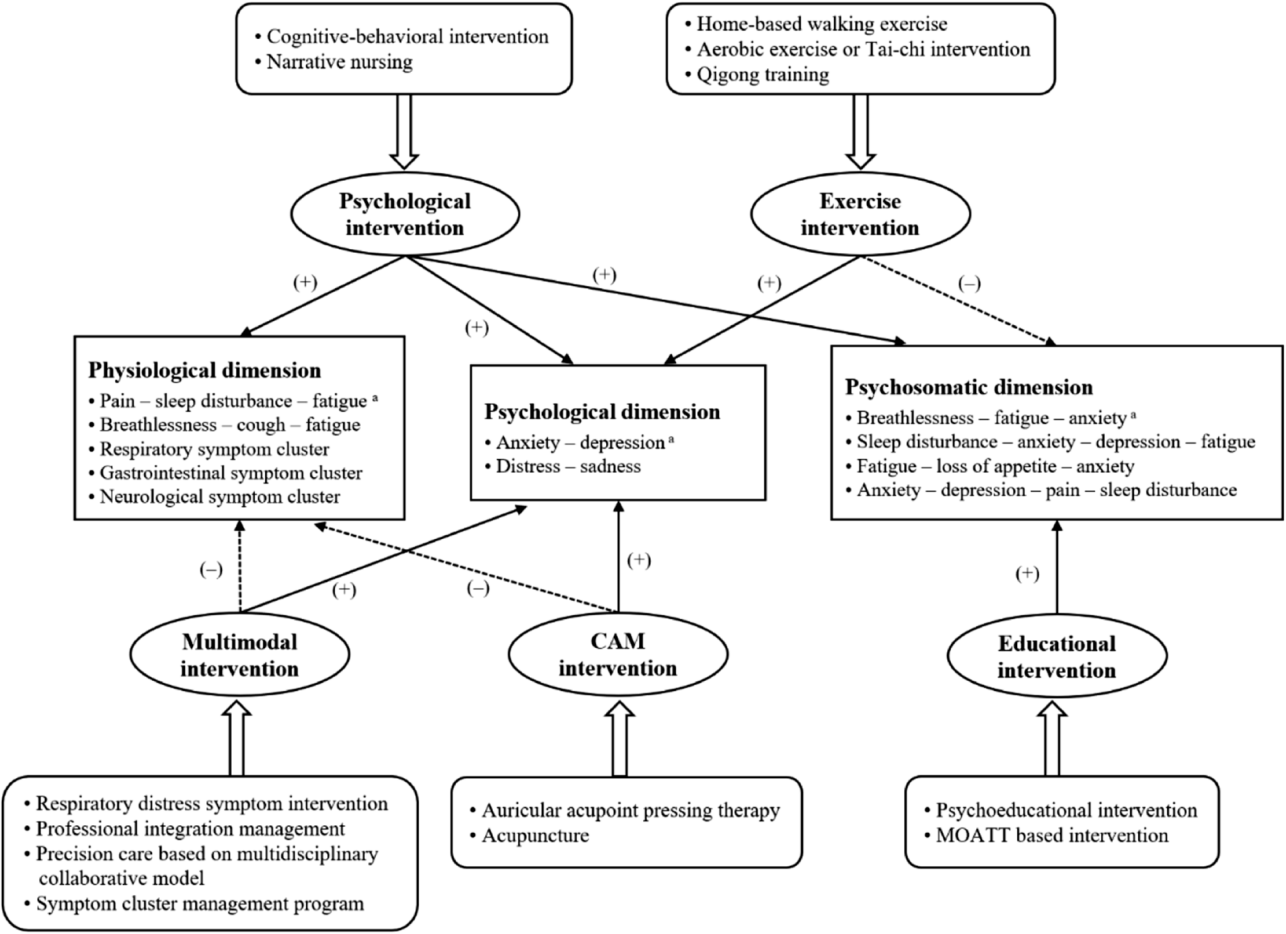
Effectiveness of the different interventions Note: ( +) = Statistically significant; ( −) = Not statistically significant; * denotes that the symptom cluster was examined most frequently in the included studies; CAM, complementary or alternative medicine; MOATT, Multinational Association for Supportive Care in Cancer Oral Agent Teaching Tool

As depicted in Table 3 and Fig. 2, we categorized symptom clusters into physiological, psychological, and psychosomatic dimensions. The most frequently assessed symptom clusters were pain-fatigue-sleep disturbance (*n* = 4), anxiety-depression (*n* = 4), and breathlessness-fatigue-anxiety (*n* = 2). Additionally, individual symptoms and other outcomes, such as QOL and functional ability, were assessed using scales or questionnaires. In the included studies, outcomes were measured initially before the intervention (baseline), and subsequently one or more times after the intervention, with two to four assessments in total. The most common time point for follow-up surveys was at the end of the intervention (*n* = 15), followed by two months (*n* = 2) and three months (*n* = 2) after completion of the intervention.

**Table 3:** Effectiveness of the different intervention types

Intervention type	Primary outcome	Secondary outcome	
	**Symptom cluster**	**Single symptom**	**Other outcomes**
Educational intervention	**• Breathlessness-fatigue-anxiety symptom cluster ** [31] *End of intervention:* Breathlessness ( +): Cohen's d = 0.64 Fatigue ( +): Cohen's d = 0.47 Anxiety ( +): Cohen's d = 1.03 *Follow-up after 3 weeks:* Breathlessness ( +): Cohen's d = 0.51 Fatigue ( +): Cohen's d = 0.64 Anxiety ( +): Cohen's d = 0.48 *Follow-up after 9 weeks:* Breathlessness ( +): Cohen's d = 0.39 Fatigue ( +): Cohen's d = 0.25 Anxiety (-)	**• Pain, fatigue, nausea, sleep disturbance, distress,** **shortness of breath, difficulty remembering, lack of** **appetite, drowsiness, dry mouth, sadness, vomiting,** **numbness ** [37] *End of intervention:* Pain ( +): Cohen's d = 1.44 Fatigue ( +): Cohen's d = 0.60 Nausea ( +): Cohen's d = 2.19 Sleep disturbance ( +): Cohen's d = 0.84 Distress ( +): Cohen's d = 1.76 Shortness of breath ( +): Cohen's d = 0.84 Difficulty remembering ( +): Cohen's d = 0.75 Lack of appetite ( +): Cohen's d = 0.91 Drowsiness ( +): Cohen's d = 0.70 Dry mouth ( +): Cohen's d = 0.72 Sadness ( +): Cohen's d = 0.91 Vomiting ( +): Cohen's d = 0.44 Numbness ( +): Cohen's d = 0.73	**• Functional Ability ** [31] *End of intervention:* - Cohen's d = 0.16 ( +) *Follow-up after 3 weeks:* - Cohen's d = 0.46 ( +) *Follow-up after 9 weeks:* - Cohen's d = 0.09 ( +)
Exercise intervention	**• Sleep disturbance-anxiety-depression-fatigue symptom cluster ** [19] *All measurement timepoints:* **Aerobic exercise group:** ● Sleep disturbance (-) ● Anxiety (-) ● Depression (-) ● Fatigue (-) **Tai-chi group:** ● Sleep disturbance (-) ● Anxiety (-) ● Depression (-) ● Fatigue (-) **• Breathlessness-fatigue-anxiety symptom cluster ** [20] *End of intervention:* ● Fatigue (-) ● Breathlessness (-) ● Anxiety (-) *Follow-up after 6 weeks:* ● Fatigue (-) ● Anxiety (-) Breathlessness ( +)	**• Pain, fatigue, nausea, sleep disturbance, sadness, shortness of breath, difficulty remembering, poor appetite, drowsiness, dry mouth, distress, vomiting and numbness ** [18]** (-)** **• Cough ** [20] - End of intervention (-) - Follow-up after 6 weeks ( +) **• Anxiety and depression ** [18] *End of intervention:* Anxiety ( +): Cohen's d = 0.19 Depression ( +): Cohen's d = 0.63 *Follow-up after 3 months:* Anxiety ( +): Cohen's d = 0.28 Depression ( +): Cohen's d = 0.42	**• Physical performance ** [18] **Aerobic exercise group:** - Time up-and-go ( +) - 30 s sit-to-stand test ( +) **• QOL, one-year survival, activity levels, circadian rhythms ** [18] (-) **• Global health status ** [19] - End of intervention (-) - Follow-up after 6 weeks ( +) **• Functional health ** [19] - End of intervention (-) - Follow-up after 6 weeks ( +) **• QOL ** [19] - End of intervention (-) - Follow-up after 6 weeks ( +)
Psychological intervention	**• Fatigue-loss of appetite-anxiety symptom cluster ** [15] *Day 7 of intervention:* Fatigue ( +): Cohen’s d = 0.89 Loss of appetite ( +): Cohen's d = 1.16 Anxiety ( +): Cohen's d = 1.32 *Day 14 of intervention:* Fatigue ( +): Cohen's d = 1.73 Loss of appetite ( +): Cohen's d = 1.58 Anxiety ( +): Cohen's d = 1.63 *End of intervention:* Fatigue ( +): Cohen's d = 2.00 Loss of appetite ( +): Cohen's d = 1.74 Anxiety ( +): Cohen's d = 1.71 **• Pain-insomnia-fatigue symptom cluster ** [35] *Three days after surgery:* Pain ( +): Cohen's d = 1.47 Insomnia ( +): Cohen's d = 1.96 Fatigue ( +): Cohen's d = 1.01 *End of intervention:* Pain ( +): Cohen's d = 1.43 Insomnia ( +): Cohen's d = 1.72 Fatigue ( +): Cohen's d = 1.63 • **Anxiety-depression-pain- sleep disturbance symptom cluster ** [16] *End of intervention:* Anxiety ( +): Cohen's d = 0.59 Depression ( +): Cohen's d = 0.61 Pain ( +): Cohen's d = 0.83 Sleep disturbance ( +): Cohen's d = 0.79	**• Anxiety and depression ** [35] *3 days after surgery:* Anxiety ( +): Cohen's d = 2.00 Depression ( +): Cohen's d = 2.14 *End of intervention:* Anxiety ( +): Cohen's d = 1.70 Depression ( +): Cohen's d = 1.78	**• Length of stay ** [35] *End of intervention:* - Cohen's d = 1.16 ( +) **• Mood state ** [15] *End of intervention:* - Cohen's d = 0.52 ( +)
Multimodal intervention	**• Breathlessness-cough-fatigue symptom cluster** *End of intervention:* Breathlessness (-) [33], ( +) Cohen's d = 1.79 [32] Cough (-) [33], ( +) Cohen's d = 3.92 [32] Fatigue (-) *Follow-up after 8 weeks:* Breathlessness ( +) [33], ( +) Cohen's d = 2.36 [32] Cough (-) [33], ( +) Cohen's d = 4.68 [32] Fatigue (-) [33], ( +) Cohen's d = 2.73 [32] **• Fatigue-pain-sleep disturbance symptom cluster** *End of intervention:* Fatigue ( +): Cohen's d = 0.60 [17]/2.05 [38] Pain ( +): Cohen's d = 1.79 [17]/0.95 [38] Sleep disturbance ( +): Cohen's d = 0.99 [17]/1.43 [38]	**• Anxiety and depression** *End of intervention:* Anxiety (-) [33], ( +): Cohen's d = 1.72 [32] Depression (-) [32, 33] *Follow-up after 8 weeks:* Anxiety (-) [32, 33] Depression (-) [32, 33] **• Pain, fatigue, nausea, sleep disturbance, distress, shortness of breath, difficulty remembering, lack of appetite, drowsiness, dry mouth, sadness, vomiting, numbness, cough, constipation, sore throat ** [36] *End of intervention:* Fatigue ( +): Cohen's d = 0.67 Sleep disturbance ( +): Cohen's d = 0.70 Distress ( +): Cohen's d = 0.79 Lack of appetite ( +): Cohen's d = 1.34 Drowsiness ( +): Cohen's d = 0.61 Sadness ( +): Cohen's d = 0.66 Cough ( +): Cohen's d = 0.56 Constipation ( +): Cohen's d = 0.57 ● Pain (-) ● Nausea (-) ● Shortness of breath (-) ● Difficulty remembering (-) ● Dry mouth (-) ● Vomiting (-) ● Numbness (-) ● Sore throat (-)	**• QOL** *End of intervention:* - (-) [31] - Cohen's d = 0.86 ( +) [36] *Follow-up after 8 weeks:* - ( +) [31]
CAM intervention	**• Five symptom clusters ( +) ** [34] *End of intervention:* Respiratory symptom cluster Pain-fatigue- sleep disturbance symptom cluster Psychological and emotional symptom cluster Gastrointestinal symptoms cluster Neurological symptom cluster **• Cough-expectoration-shortness of breath symptom cluster ** [39] *End of intervention:* Cough ( +): Cohen's d = 2.92 Expectoration (-): Cohen's d = 1.94 Shortness of breath (-): Cohen's d = 2.28	NR	**• QOL** *End of intervention:* *-* Cohen's d = 1.17 ( +) [38]

*Note: NR* not reported, *QOL* quality of life, *CAM* complementary or alternative medicine

#### Educational intervention

Two studies [31, 37] reported that the educational interventions had a positive effect on the management of the breathlessness-fatigue-anxiety symptom cluster in lung cancer patients. Moderate to substantial effects of the intervention were observed for this symptom cluster (Cohen's d ranged from 0.47 to 1.03), and positive effects persisted for three weeks and nine weeks after the intervention. Among single symptoms, the most significant post-intervention improvement was observed for nausea (Cohen's d = 2.19), followed by distress (Cohen's d = 1.76) and then pain (Cohen's d = 1.44). Furthermore, the educational intervention resulted in a significant improvement in functional ability that lasted for nine weeks after the intervention (Cohen's d ranged from 0.09 to 0.46).

#### Exercise intervention

In three studies [18–20], exercise interventions were primarily employed to manage the sleep disturbance-anxiety-depression-fatigue symptom cluster and the breathlessness-fatigue-anxiety symptom cluster. However, no significant improvement was observed in these symptom clusters post-intervention. With respect to individual symptoms, the findings were mixed. One study found that an exercise intervention had a moderate effect on the improvement of anxiety and depression that lasted for three months (Cohen's d ranged from 0.19 to 0.63), but the impact on physical symptoms was insignificant (*p* > 0.05). Conversely, another study reported a notable improvement in cough following a six-week follow-up (*p* < 0.05). Additionally, there were significant enhancements in global health, functional health, and symptom-related QOL at six weeks post-intervention (*p* < 0.05).

#### Psychological intervention

All three studies included [15, 16, 35] demonstrated favorable effects of psychological interventions on the following symptom clusters: fatigue-loss of appetite-anxiety cluster (Cohen's d from 1.49 to 1.55), pain-insomnia-fatigue cluster (Cohen's d ranged from 1.32 to 1.84), and anxiety-depression-pain-sleep disturbance symptom cluster (Cohen's d ranged from 0.59 to 0.83). However, the sustained effects of these intervention have not been reported. In terms of individual symptoms, psychological interventions have been reported to have a strong effect on alleviating anxiety and depression in lung cancer patients (Cohen's d = 1.85 and 1.96, respectively). Additionally, patients who received psychological interventions experienced a shorter length of stay (Cohen's d = 1.16) and reported better mood states (Cohen's d = 0.52) than did those in the control group.

#### Multimodal intervention

Among the five multimodal interventions [17, 32, 33, 36, 38], two studies reported significant improvements in the fatigue-pain-sleep disturbance symptom cluster, with substantial effect sizes (Cohen's d ranging from 0.60 to 2.05). However, the effects on the breathlessness-cough-fatigue symptom cluster were mixed; one study reported a large effect of the multimodal intervention (Cohen's d ranging from 1.79 to 4.68), whereas the other study reported insignificant findings. With respect to individual symptoms, the multimodal intervention was found to be significant for only half of the 16 symptoms evaluated. In terms of effect size, the top three symptoms were lack of appetite (Cohen's d = 1.34) and distress (Cohen's d = 0.79), followed by sleep disturbance (Cohen's d = 0.70). Furthermore, mixed findings regarding anxiety, depression, and QOL have been reported.

#### CAM intervention

Only two of the studies included in this review utilized a CAM intervention. Jiang et al. [34] used auricular acupressure therapy, which targets symptom clusters, including respiratory, pain-fatigue-sleep disturbance, psychological and emotional, gastrointestinal, and neurological symptom clusters. The study demonstrated significant improvement in all five symptom clusters and in QOL (Cohen's d = 1.17) within the intervention group, with a statistically significant difference from the control group. In addition, Zhang et al. [39] verified the efficacy of acupuncture on a cough-related symptom cluster, and the results showed that acupuncture could relieve the cough of lung cancer patients but did not relieve expectoration or shortness of breath. However, the specific effects of CAM interventions remain unclear due to the absence of similar studies at this time.

### Effectiveness of interventions on single symptoms

In this review, meta-analyses were conducted to explore the effects of non-pharmacological interventions on individual symptoms. The outcome indicators were centred on the four most frequently mentioned symptoms in the studies: fatigue (*n* = 10), anxiety (*n* = 6), sleep disturbance (*n* = 5), and depression (*n* = 5). Importantly, the CAM intervention study was not included in the meta-analysis because of a lack of single symptom scores.

#### Fatigue

**Fig. 3:**
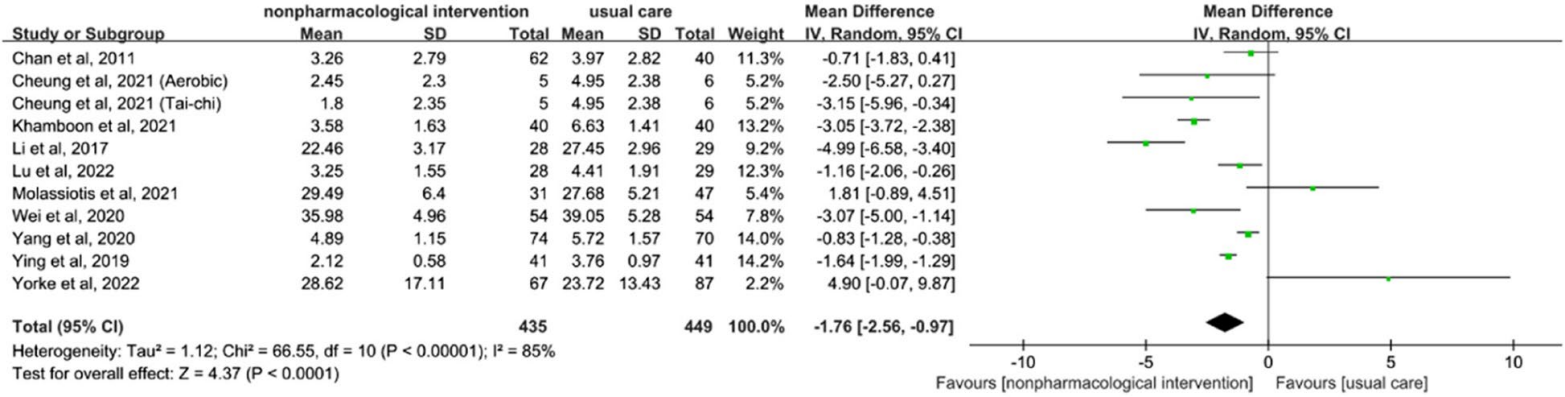
Forest plot of non-pharmacological interventions on fatigue

**Fig. 4:**
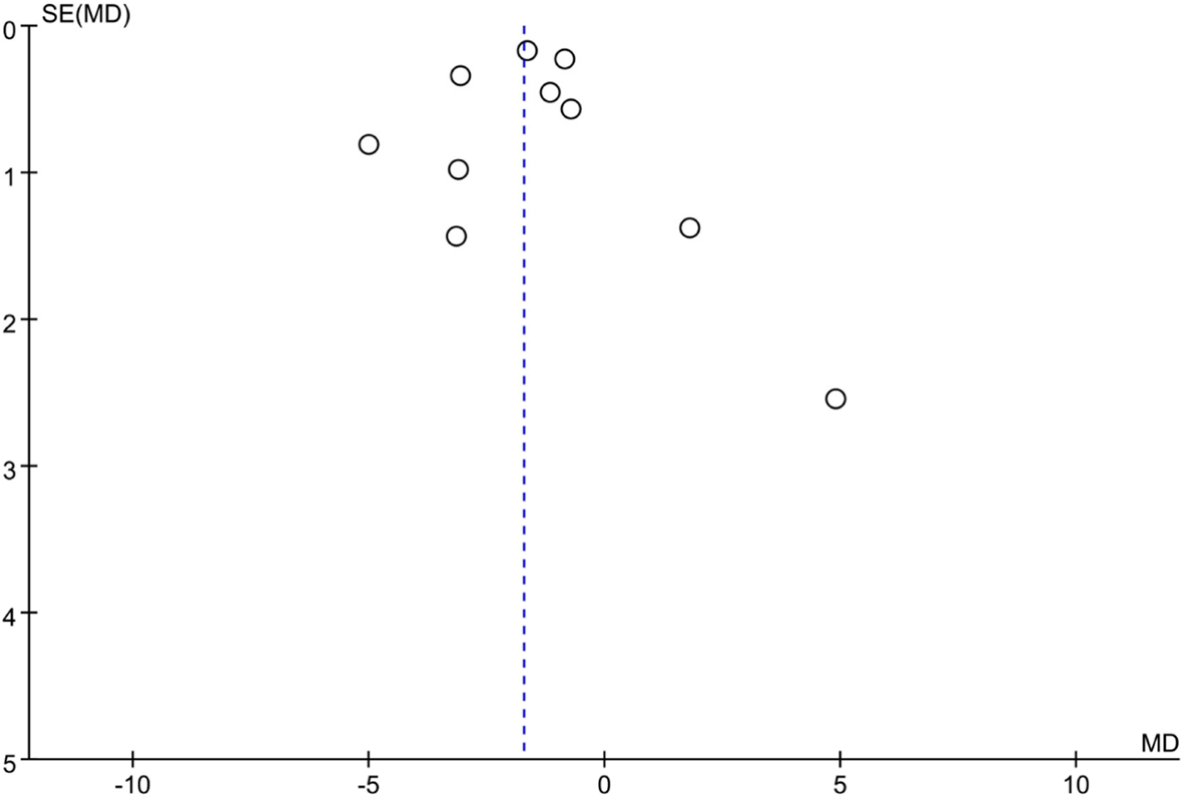
Funnel plot of fatigue

The synthesis of ten studies, involving a total of 878 patients, demonstrated statistically significant effects of the non-pharmacological interventions on fatigue (SMD = -1.76, 95% CI [-2.56, -0.97], *p* < 0.01; see Fig. 3). A random-effects model was applied because statistically significant heterogeneity was observed (I^2^ = 85%). Sensitivity analyses indicated that excluding any of the studies did not substantially decrease the overall heterogeneity. Subgroup analyses revealed that different types of non-pharmacological interventions had varying effects on fatigue. Specifically, only the educational intervention subgroup [31, 37] exhibited low heterogeneity and proved effective in improving fatigue (SMD = -0.81, 95% CI [-1.23, -0.39], *p* < 0.01, I^2^ = 0%; refer to Appendix 4). Among the ten included studies, no statistical (Egger's test, *p* = 0.916) or visual (Fig. 4) evidence of publication bias was detected.

#### Anxiety

**Fig. 5:**
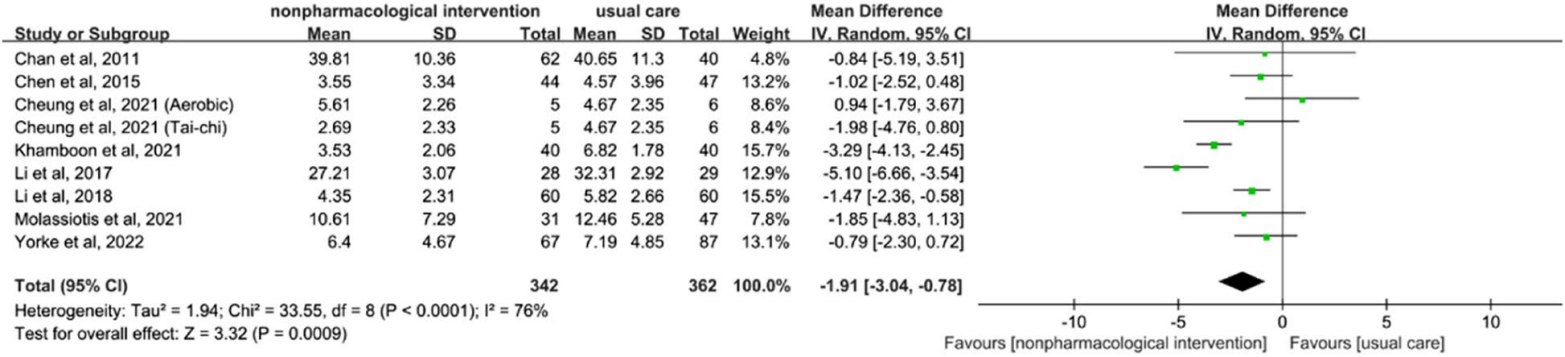
Forest plot of non-pharmacological interventions on anxiety

Six studies, encompassing a total of 698 patients, investigated the effects of non-pharmacological interventions on anxiety. The meta-analysis revealed a statistically significant effect (SMD = -1.91, 95% CI [-3.04, -0.78], *p* < 0.01; see Fig. 5). Given the high heterogeneity (I^2^ = 76%), a random-effects model was employed. Sensitivity analysis indicated that excluding any single study did not substantially alter the pooled heterogeneity. Subgroup analysis revealed that although psychological interventions were effective in alleviating anxiety, heterogeneity persisted (SMD = -3.19, 95% CI [-5.02, -1.37],* p* < 0.01, I^2^ = 89%; refer to Appendix 5).

#### Sleep sisturbance

**Fig. 6:**
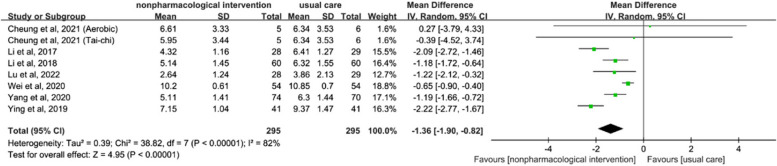
Forest plot of non-pharmacological interventions on sleep disturbance

The meta-analysis of sleep disturbance included five studies involving 424 lung cancer patients. The results revealed statistically significant effects of the non-pharmacological interventions (SMD = -1.36, 95% CI [-1.90, -0.82], *p* < 0.01; see Fig. 6). Given the presence of statistically significant heterogeneity (I^2^ = 82%), a random-effects model was employed. In the sensitivity analysis, excluding any single study did not substantially modify the overall heterogeneity. Subsequent subgroup analyses were performed to explore the effects of different non-pharmacological interventions on sleep disturbance. Nevertheless, both the psychological intervention subgroup and the multimodal intervention subgroup retained significant heterogeneity (I^2^ = 78% and 92%, respectively; see Appendix 6).

#### Depression

**Fig. 7:**
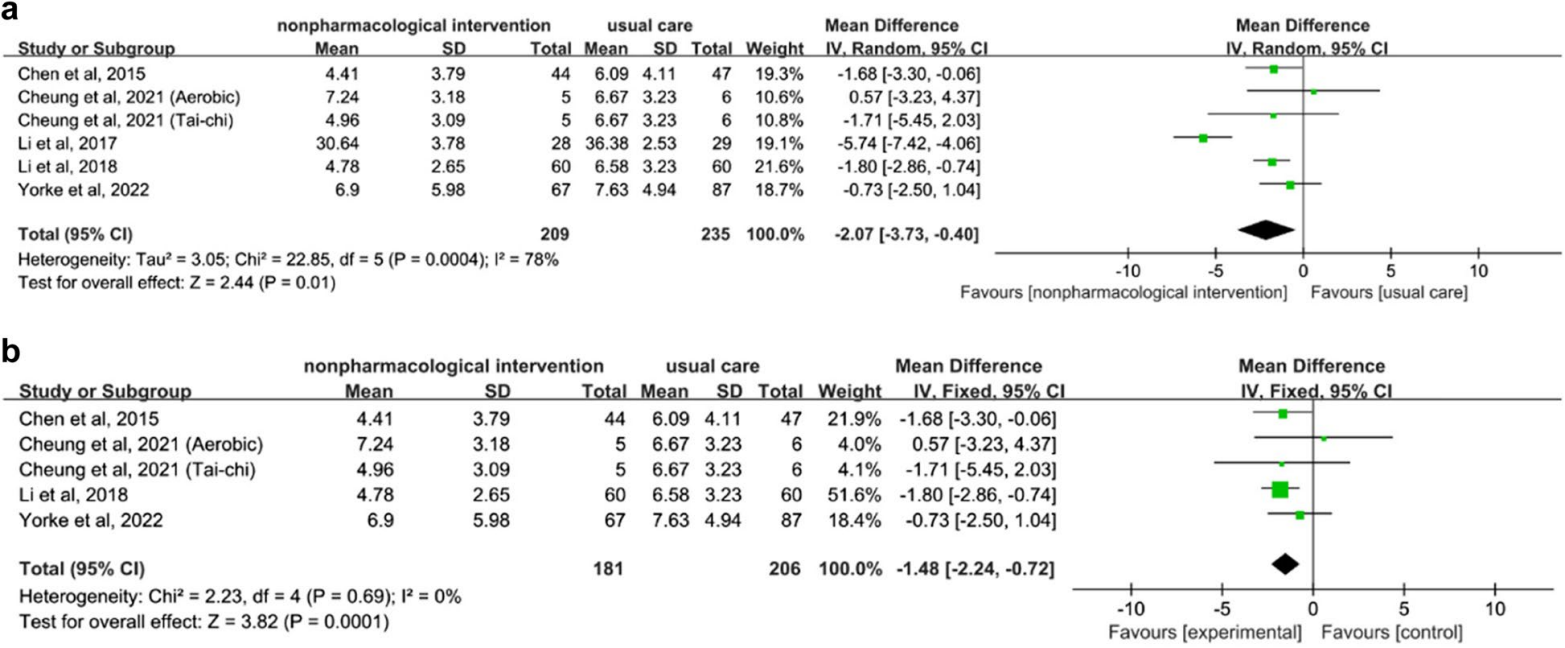
**a** Forest plot of non-pharmacological interventions on depression. **b** Sensitivity analysis of non-pharmacological interventions on depression

Five studies, involving 438 patients, investigated the impact of non-pharmacological interventions on depression. The results demonstrated statistically significant effects (SMD = -2.07, 95% CI [-3.73, -0.40], *p* < 0.01; see Fig. 7a). Owing to the presence of statistically significant heterogeneity (I^2^ = 78%), a random-effects model was employed. To assess the influence of individual studies on heterogeneity, a sensitivity analysis was conducted by sequentially excluding each study. After excluding the study by Li et al. [35], the remaining four studies exhibited acceptable heterogeneity (I^2^ = 0%). Consequently, a fixed-effects model was utilized. Pooled effects indicated that non-pharmacological interventions had a positive impact on depression compared with the control conditions (SMD = -1.48, 95% CI [-2.24 -0.72], *p* < 0.01; see Fig. 7b).

## Discussion

This systematic review offers initial insights into the effectiveness of non-pharmacological interventions in managing symptom clusters among lung cancer patients. This analysis suggests that psychological and educational interventions effectively mitigate the severity of most symptom clusters. Particularly noteworthy is the significant reduction in severity observed in the pain-sleep disturbance-fatigue and fatigue-loss of appetite-anxiety symptom clusters [15, 35]. However, the effectiveness of exercise-based, multimodal, and CAM interventions remains inconclusive. For instance, mixed results were noted for clusters such as breathlessness-cough-fatigue and breathlessness-fatigue-anxiety [20, 32, 33]. These findings underscore the potential advantages of non-pharmacological interventions in addressing challenging symptom experiences in lung cancer patients, both during and after treatment. Furthermore, they emphasize the urgent need for additional interventions capable of effectively managing these symptom clusters across diverse lung cancer populations.

This study's results align with those of previous systematic reviews and meta-analyses conducted on other cancer patients [23, 40, 41] that demonstrated that educational and psychological interventions yield moderate to large effect sizes for symptom clusters and functional abilities. Moreover, this meta-analysis found significant improvements in fatigue, anxiety, sleep disturbance and depression in the intervention groups. These findings underscore the effectiveness of interventions that include education for lung cancer patients regarding the nature of their experienced symptoms, equipping them with essential self-care knowledge, skills, confidence, and motivation. Furthermore, interventions employing psychological techniques to manage symptom clusters have also proven beneficial. The Guidelines for Holistic Integrative Management of Cancer, published by the Chinese Anti-Cancer Association in 2022, recommend implementing cognitive-behavioural therapy to improve symptoms such as anxiety, depression, and sleep disturbance in cancer patients, and utilizing patient education to alleviate cancer-related fatigue [42].

Contrary to findings in other reviews of cancer patients [22, 43], exercise interventions, as adopted in three studies, appeared to have no significant effect on symptom clusters among lung cancer patients. They showed significant changes only for certain individual symptoms, such as breathlessness, anxiety, and depression. According to international guidelines [44], exercise interventions require a specific duration and intensity to yield a significant effect. Compared with other cancer populations, lung cancer patients often experience more severe symptoms, have limited mobility, and have a reduced tolerance for exercise [45]. This may hinder their ability to reach the necessary thresholds for effective exercise interventions. Furthermore, long-term follow-up observations are necessary to assess the enduring impact of exercise on health outcomes.

Compared with other interventions, CAM interventions were introduced relatively late, and current evidence has yielded inconclusive results. Our review findings indicated that auricular acupressure therapy led to significant improvements in five symptom clusters and in the QOL of patients with lung cancer, which aligns with the findings of a systematic review assessing the effectiveness of acupressure on fatigue-related symptom clusters in patients with breast cancer [23]. However, acupuncture was only meaningful for cough in the cough-expectoration-shortness symptom cluster, which may be because the intervention was too short to show long-term effects. Importantly, only two CAM intervention studies were included in our review. Therefore, further investigations into the effectiveness of CAM interventions in managing symptom clusters in lung cancer patients are warranted.

Similarly, drawing specific conclusions about the effects of multimodal interventions remains challenging. While multimodal interventions significantly improved the fatigue-pain-sleep disturbance symptom cluster and QOL, the effect sizes ranged from moderate to large. However, for individual symptoms, meta-analyses revealed a significant effect of multimodal interventions on sleep disturbance, but not on fatigue. This aligns with the findings of the systematic review conducted by So et al. [21], which indicated a low strength of evidence for multimodal interventions. Two possible reasons account for this result. First, the diverse forms of multimodal interventions lead to a high degree of methodological heterogeneity between studies, making it challenging to arrive at definitive conclusions. Second, the sample sizes were insufficient; for example, Yorke et al. did not observe a significant improvement in the symptom cluster in their feasibility randomized trial [33], whereas such an improvement was later noted in a multicentre, large-sample RCT [32].

### Review impact

There are some caveats to this review that may direct recommendations for future research and practice in symptom cluster interventions for lung cancer patients. First, the 15 studies that were included employed a wide variety of interventions that targeted different symptom clusters. Therefore, it is likely that the content of the reported interventions may not fully address certain symptom clusters examined in the studies. All 15 interventions utilized a face-to-face format, with only 27% (*n* = 4) employing a combination of individual and group approaches. Given the advancements in internet technology and big data platforms, future interventions can leverage diversified and blended online-offline approaches. Real-time monitoring and feedback on symptom management effectiveness through mobile apps or platforms like WeChat groups can enhance the intervention process. These tools can improve the efficiency and compliance of patient self-management, ultimately optimizing outcomes [46].

Second, fewer than 33% (*n* = 5) of the studies incorporated theories, models, or frameworks into the development of interventions, and these theoretical foundations varied widely. A robust theory, model, or framework proves invaluable in crafting rigorous interventions, establishing measurements and outcomes, and elucidating the mechanisms through which interventions operate [47]. Therefore, we encourage future studies to adopt specific theories or frameworks related to symptom management to guide study design and implementation. This approach can provide a deeper understanding of the nature, progression, and management strategies of symptoms, ensuring that research becomes more systematic, scientific, and feasible.

Third, the heterogeneity of the participants may have contributed to the observed discrepancies in reported intervention effects. Differences in lung cancer staging and treatment types not only influence symptom severity but also lead to variations in other outcomes, such as QOL and functional ability [48, 49]. Among the 15 studies included, the majority of patients were in advanced stages of lung cancer and were receiving treatments such as chemotherapy or surgery. This may be due to the greater symptom complexity and treatment challenges faced by patients with advanced lung cancer. Only a few studies have developed interventions specifically tailored to address symptom clusters among those with early-to-mid-stage lung cancers or those who are no longer undergoing active treatment. However, individuals with lung cancer in treatment intermission or those receiving home care may experience a range of symptom clusters and may benefit from non-pharmacological interventions to help manage them [5, 50]. This highlights the pressing need for more symptom cluster interventions tailored to various types of lung cancer, addressing unmet health needs at different stages of survivorship. Focusing on early- and middle-stage patients and exploring the effects of non-pharmacological interventions will help comprehensively understand and optimize the whole-cycle management of lung cancer patients.

Finally, the studies included in this review primarily focused on symptom cluster severity, QOL, and functional ability among intervention participants, employing a wide range of assessment tools. Other important dimensions—such as the timing, distress, and quality of symptom clusters—as well as indicators like patient morbidity and mortality, were largely overlooked. While our meta-analysis revealed significant improvements in individual symptoms, including fatigue, anxiety, sleep disturbance, and depression within the intervention groups, it is important to acknowledge the potential presence of heterogeneity. Differences in intervention measures also contributed to this heterogeneity. This suggests that, with a larger number of future intervention studies, subgroup analyses in systematic reviews could help further refine the sources of heterogeneity. Additionally, variations in data collection time points made it challenging to draw direct comparisons between the study results.The interventions described in the 15 studies all had relatively short durations, with the longest reported intervention lasting only 12 weeks. As such, there is a general lack of evidence regarding the effectiveness of long-term non-pharmacological interventions. Therefore, future research should focus on longer-term interventions with additional follow-up assessments of patient outcomes, potentially providing valuable data on the optimal duration for particular interventions to achieve maximum beneficial effects.

### Limitations

This review is subject to several limitations. First, the review considered only RCTs and quasi-experimental studies, excluding cohort studies or self-control case studies, which could have led to the oversight of important findings. Second, the inclusion criteria were limited to studies published in English or Chinese, possibly excluding eligible studies in other languages and introducing a language bias. Third, the relatively small number of studies included in this review may have excluded interventions with controversial effectiveness, making definitive conclusions difficult to draw. This also limits the generalizability of the meta-analysis results, and heterogeneity cannot be accurately assessed. Consequently, more high-quality, large-sample studies are needed in the future to verify and expand our findings. Finally, the findings of this study primarily reflect the Asian population; future research involving diverse ethnic populations could enhance the applicability of the results.

### Implications

Previous systematic reviews have focused on overall cancer or breast cancer populations and have focused mostly on specific symptom clusters, with insufficient attention given to the management of symptom clusters in patients with lung cancer. This review concentrated on the management of symptom clusters in lung cancer patients, thus filling this research gap, and contributing to oncology-related nursing research and practice. The studies included in this review reported a wide range of non-pharmacological interventions that could benefit lung cancer patients in terms of symptom management and improvements in QOL. Our study suggests that educational and psychological interventions are the most effective in managing symptom clusters in lung cancer patients, which provides direction for healthcare professionals to develop management programs. Future research should build on our findings to strengthen the evidence supporting the effectiveness of exercise and multimodal interventions, as well as their applicability in clinical settings.

## Conclusions

While research on symptom clusters in lung cancer symptom management has received significant emphasis, studies testing interventions specifically targeting core symptom clusters are notably scarce. Psychological and educational interventions have demonstrated efficacy in managing symptom clusters in lung cancer patients. However, the effects of exercise, multimodal approaches and CAM interventions require further exploration. Hence, in forthcoming studies, it is imperative to design evidence-based and rigorous intervention methods tailored for core or sentinel symptom clusters, particularly for vulnerable lung cancer patients at varying stages of survivorship.

## Supplementary Information

Supplementary Material 1.

## Data Availability

No datasets were generated or analysed during the current study.

## Abbreviations

QOL: Quality of life
MOATT: Multinational association for supportive care in cancer oral agent teaching tool
CAM: Complementary or alternative medicine
