# Supplementary material for: Effectiveness of non-pharmacological interventions in managing symptom clusters among lung cancer patients: a systematic review
Source: BMC Cancer. 2024 Dec 6;24:1505. doi: 10.1186/s12885-024-13246-x (PMC11622468; doi:10.1186/s12885-024-13246-x)
Supplement: Supplementary file 1 — Supplementary Material 1. [file 12885_2024_13246_MOESM1_ESM.docx]

| **Search** | **Search strategy** | **Result** |
| --- | --- | --- |
| #1 | "Lung Neoplasms"[Mesh] OR "lung neoplas*"[Title/Abstract] OR "lung cancer*"[Title/Abstract] OR "lung carcinoma*"[Title/Abstract] OR "lung tumo*"[Title/Abstract] OR "lung malignan*"[Title/Abstract] | 349938 |
| #2 | "Syndrome"[Mesh] OR "symptom cluster*"[Title/Abstract] OR "multiple symptom*"[Title/Abstract] OR "symptom constellation*"[Title/Abstract] OR "concurrent symptom*"[Title/Abstract] OR "co-occurring symptom*"[Title/Abstract] | 127545 |
| #3 | #1 AND #2 | 761 |

**Appendix 1.** The search strategy for PubMed

| **Bias domain** | **Signaling questions** | **Option** | **Wei et al. 2020** | **Jiang et al.**  **2022** | **Zhang**  **2022** | **Feng et al.**  **2020** | **Ying et al.**  **2019** | **Yang et al.**  **2020** | **Pang 2021** | **Yu et al.**  **2022** | **Liu et al.**  **2020** | **Sun et al.**  **2019** | **Zhao et al.**  **2022** | **Jiao et al.**  **2022** |
| --- | --- | --- | --- | --- | --- | --- | --- | --- | --- | --- | --- | --- | --- | --- |
| Bias arising  from the  randomization  process | 1.1 Was the allocation sequence random? | Y/PY/PN/N/NI | Y | Y | Y | N | PY | Y | Y | Y | PY | Y | PY | Y |
|  | 1.2 Was the allocation sequence concealed until participants were recruited and assigned to interventions? | Y/PY/PN/N/NI | NI | NI | NI | PN | NI | NI | NI | NI | NI | NI | NI | NI |
|  | 1.3 Were there baseline imbalances that suggest a problem with the randomization process? | Y/PY/PN/N/NI | N | N | N | N | N | N | N | N | N | N | PN | N |
|  |  |  | **Low** | **Low** | **Low** | **High** | **Some concern** | **Low** | **Low** | **Low** | **Some concern** | **Low** | **Some concern** | **Low** |
| Bias due to  deviations  from intended  interventions | 2.1 Were participants aware of their assigned intervention during the trial? | Y/PY/PN/N/NI | N | N | N | N | N | N | N | N | N | N | N | N |
|  | 2.2 Were carers and trial personnel aware of participants' assigned intervention during the trial? | Y/PY/PN/N/NI | Y | Y | Y | Y | Y | Y | Y | Y | Y | Y | Y | Y |
|  | 2.3 If Y/PY/NI to 2.1 or 2.2: Were there deviations from the intended intervention beyond what would be expected in usual practice? | NA/Y/PY/PN/N/NI | NI | Y | NI | NI | NI | NI | NI | NI | NI | NI | NI | NI |
|  | 2.4. If Y/PY to 2.3: Were these deviations from intended intervention unbalanced between groups and likely to have affected the outcome? | NA/Y/PY/PN/N/NI |  | N |  |  |  |  |  |  |  |  |  |  |
|  | 2.5 If Y/PY/NI to 2.4: Did the deviation from established intervention measures occur equally across groups? | NA/Y/PY/PN/N/NI |  |  |  |  |  |  |  |  |  |  |  |  |
|  | 2.6 Was an appropriate analytical method used to estimate the effect of intervention measure allocation? | NA/Y/PY/PN/N/NI | Y | Y | Y | Y | Y | Y | Y | Y | Y | Y | Y | Y |
|  | 2.7 If N/PN/NI to 2.6: Was there a potential for significant impact on the results due to misclassification of subjects during group analysis? | NA/Y/PY/PN/N/NI |  |  |  |  |  |  |  |  |  |  |  |  |
|  |  |  | **Some concern** | **Some concern** | **Some concern** | **Some concern** | **Some concern** | **Some concern** | **Some**  **concern** | **Some concern** | **Some concern** | **Some concern** | **Some concern** | **Some concern** |
| Bias due to deviating from established intervention measures (the effect of compliance with intervention measures) | 2.1 Were participants aware of their assigned intervention during the trial? | NA/Y/PY/PN/N/NI | Y | Y | Y | Y | Y | Y | Y | Y | Y | Y | Y | Y |
|  | 2.2 Were carers and trial personnel aware of participants' assigned intervention during the trial? | NA/Y/PY/PN/N/NI | Y | Y | Y | Y | Y | Y | Y | Y | Y | Y | Y | Y |
|  | 2.3 [If applicable] If Y/PY/NI to 2.1 or 2.2, were important unplanned interventions balanced between the groups? | NA/Y/PY/PN/N/NI | Y | Y | Y | Y | Y | Y | Y | Y | Y | Y | Y | Y |
|  | 2.4 [If applicable] Could the non-completion of the intervention affect the outcome? | NA/Y/PY/PN/N/NI | N | N | N | N | N | N | N | N | N | N | N | N |
|  | 2.5 [If applicable] Could non-compliance with intervention measures potentially affect the outcomes of the participants? | NA/Y/PY/PN/N/NI | NA | NA | NA | NA | NA | NA | NA | NA | NA | NA | NA | NA |
|  | 2.6 If N/PN/NT to 2.3. or Y/PY/NI to 2.4 or 2.5: Could non-compliance with intervention measures potentially affect the outcomes of the participants? | NA/Y/PY/PN/N/NI |  |  |  |  |  |  |  |  |  |  |  |  |
|  |  |  | **Low** | **Low** | **Low** | **Low** | **Low** | **Low** | **Low** | **Low** | **Low** | **Low** | **Low** | **Low** |
| Bias due to  missing  outcome data | 3.1 Were outcome data available for all, or nearly all? | NA/Y/PY/PN/N/NI | Y | Y | Y | NI | Y | Y | Y | Y | Y | Y | Y | Y |
|  | 3.2 If N/PN/NI to 3.1: Is there evidence that results were robust to the presence of missing outcome data? | NA/Y/PY/PN/N/NI |  |  |  | N |  |  |  |  |  |  |  |  |
|  | 3.3 If N/PN to 3.2: Is it possible for missing data to depend on its true value? | NA/Y/PY/PN/N/NI |  |  |  | NI |  |  |  |  |  |  |  |  |
|  | 3.4 If Y/PY/NI to 3.3: Is the proportion of missing values in the outcome variable different between the two groups? | NA/Y/PY/PN/N/NI |  |  |  | NI |  |  |  |  |  |  |  |  |
|  |  |  | **Low** | **Low** | **Low** | **High** | **Low** | **Low** | **Low** | **Low** | **Low** | **Low** | **Low** | **Low** |
| Bias in  measurement  of the  outcome | 4.1 Was the outcome measurement method inappropriate? | NA/Y/PY/PN/N/NI | N | N | N | N | N | N | N | N | N | N | N | N |
|  | 4.2 Was it possible for there to be inter-group differences in outcome measurement or determination? | NA/Y/PY/PN/N/NI | N | N | N | N | N | N | N | N | N | N | N | N |
|  | 4.3 If N/PN/NI to 4.1: Did the outcome assessors know which intervention measures the subjects have received? | NA/Y/PY/PN/N/NI | Y | Y | Y | Y | Y | Y | Y | Y | Y | Y | Y | Y |
|  | 4.4 If Y/PY/NI to 4.3: Is it possible for the type of intervention received to affect the outcome measurement? | NA/Y/PY/PN/N/NI | PN | PN | PN | PN | PN | PN | PN | PN | PN | PN | PN | PN |
|  | 4.5 If Y/PY/NI to 4.4: Did knowing the intervention measures affect the measurement of outcome variables? | NA/Y/PY/PN/N/NI |  |  |  |  |  |  |  |  |  |  |  |  |
|  |  |  | **Low** | **Low** | **Low** | **Low** | **Low** | **Low** | **Low** | **Low** | **Low** | **Low** | **Low** | **Low** |
| Bias in  selection of  the reported  result | 5.1 Whether the data analysis of the results is consistent with the pre-determined analysis plan made before unblinding the outcome data? | NA/Y/PY/PN/N/NI | Y | Y | Y | Y | Y | Y | Y | Y | Y | Y | Y | Y |
|  | 5.2 Multiple outcome measurements (e.g. scales, definitions, time points) within the outcome domain? | NA/Y/PY/PN/N/NI | N | N | Y | N | N | N | Y | Y | Y | Y | Y | Y |
|  | 5.3 Multiple analyses of the data? | NA/Y/PY/PN/N/NI | N | N | N | N | N | N | N | N | N | N | N | N |
|  |  |  | **Low** | **Low** | **High** | **Low** | **Low** | **Low** | **High** | **High** | **High** | **High** | **High** | **High** |
| **Overall bias** |  |  | **Some concern** | **Some concern** | **High** | **High** | **Some concern** | **Some concern** | **High** | **High** | **High** | **High** | **High** | **High** |
| Note: Y (Yes); PY (Probably Yes); N (No); PN (Probably No); NI (No Information); NA (Not Applicable). | | | | | | | | | | | | | | |

**Appendix 2.** Bias analysis results using version 2 of the Cochrane Risk-of-Bias tool for (RoB 2) for RCTs

| **Bias domain** | **Signaling questions** | **Option** | **Li et al.**  **2018** | **Zheng et al.**  **2020** | **Lu et al.**  **2022** | **Xu et al.**  **2022** | **Cheung et al.**  **2021** | **Yorke et al.**  **2015** | **Chan et al.**  **2011** | **Chen et al.**  **2015** | **Yorke et al.**  **2022** | **Molassiotis et al.**  **2021** |
| --- | --- | --- | --- | --- | --- | --- | --- | --- | --- | --- | --- | --- |
| Bias arising  from the  randomization  process | 1.1 Was the allocation sequence random? | Y/PY/PN/N/NI | Y | PY | PY | Y | Y | Y | Y | Y | Y | Y |
|  | 1.2 Was the allocation sequence concealed until participants were recruited and assigned to interventions? | Y/PY/PN/N/NI | NI | NI | NI | NI | Y | NI | NI | Y | Y | Y |
|  | 1.3 Were there baseline imbalances that suggest a problem with the randomization process? | Y/PY/PN/N/NI | N | N | N | NI | N | PY | NI | N | NI | PN |
|  |  |  | **Low** | **Some concern** | **Some concern** | **Low** | **Low** | **Some concern** | **Low** | **Low** | **Low** | **Low** |
| Bias due to  deviations  from intended  interventions | 2.1 Were participants aware of their assigned intervention during the trial? | Y/PY/PN/N/NI | N | N | N | N | N | N | N | N | N | N |
|  | 2.2 Were carers and trial personnel aware of participants' assigned intervention during the trial? | Y/PY/PN/N/NI | Y | Y | Y | Y | Y | Y | Y | Y | Y | Y |
|  | 2.3 If Y/PY/NI to 2.1 or 2.2: Were there deviations from the intended intervention beyond what would be expected in usual practice? | NA/Y/PY/PN/N/NI | PN | NI | NI | NI | PN | PN | PN | PN | PN | PN |
|  | 2.4. If Y/PY to 2.3: Were these deviations from intended intervention unbalanced between groups and likely to have affected the outcome? | NA/Y/PY/PN/N/NI |  |  |  |  |  |  |  |  |  |  |
|  | 2.5 If Y/PY/NI to 2.4: Did the deviation from established intervention measures occur equally across groups? | NA/Y/PY/PN/N/NI |  |  |  |  |  |  |  |  |  |  |
|  | 2.6 Was an appropriate analytical method used to estimate the effect of intervention measure allocation? | NA/Y/PY/PN/N/NI | Y | Y | Y | Y | Y | Y | Y | Y | Y | Y |
|  | 2.7 If N/PN/NI to 2.6: Was there a potential for significant impact on the results due to misclassification of subjects during group analysis? | NA/Y/PY/PN/N/NI |  |  |  |  |  |  |  |  |  |  |
|  |  |  | **Low** | **Some concern** | **Some concern** | **Some concern** | **Low** | **Low** | **Low** | **Low** | **Low** | **Low** |
| Bias due to deviating from established intervention measures (the effect of compliance with intervention measures) | 2.1 Were participants aware of their assigned intervention during the trial? | NA/Y/PY/PN/N/NI | Y | Y | Y | Y | Y | Y | Y | Y | Y | Y |
|  | 2.2 Were carers and trial personnel aware of participants' assigned intervention during the trial? | NA/Y/PY/PN/N/NI | Y | Y | Y | Y | Y | Y | Y | Y | Y | Y |
|  | 2.3 [If applicable] if Y/PY/NI to 2.1 or 2.2, were important unplanned interventions balanced between the groups? | NA/Y/PY/PN/N/NI | Y | Y | Y | Y | Y | Y | Y | Y | Y | Y |
|  | 2.4 [If applicable] could the non-completion of the intervention affect the outcome? | NA/Y/PY/PN/N/NI | N | N | N | N | N | N | N | N | N | N |
|  | 2.5 [If applicable] could non-compliance with intervention measures potentially affect the outcomes of the participants? | NA/Y/PY/PN/N/NI | NA | NA | NA | NA | NA | NA | NA | NA | NA | NA |
|  | 2.6 If N/PN/NT to 3.3 or Y/PY/NI to 3.4 or 3.5: Could non-compliance with intervention measures potentially affect the outcomes of the participants? | NA/Y/PY/PN/N/NI |  |  |  |  |  |  |  |  |  |  |
|  |  |  | **Low** | **Low** | **Low** | **Low** | **Low** | **Low** | **Low** | **Low** | **Low** | **Low** |
| Bias due to  missing  outcome data | 3.1 Were outcome data available for all, or nearly all? | NA/Y/PY/PN/N/NI | Y | Y | N | Y | Y | N | N | Y | N | N |
|  | 3.2 If N/PN/NI to 3.1: Is there evidence that results were robust to the presence of missing outcome data? | NA/Y/PY/PN/N/NI |  |  | N |  |  | N | N |  | N | N |
|  | 3.3 If N/PN to 3.2: Is it possible for missing data to depend on its true value? | NA/Y/PY/PN/N/NI |  |  | NI |  |  | NI | PN |  | PN | PN |
|  | 3.4 If Y/PY/NI to 3.3: Is the proportion of missing values in the outcome variable different between the two groups? | NA/Y/PY/PN/N/NI |  |  | N |  |  | N |  |  |  |  |
|  |  |  | **Low** | **Low** | **Some concern** | **Low** | **Low** | **Some concern** | **Low** | **Low** | **Low** | **Low** |
| Bias in  measurement  of the  outcome | 4.1 Was the outcome measurement method inappropriate? | NA/Y/PY/PN/N/NI | N | N | N | N | N | N | N | N | N | N |
|  | 4.2 Was it possible for there to be inter-group differences in outcome measurement or determination? | NA/Y/PY/PN/N/NI | N | N | N | N | N | N | N | N | N | N |
|  | 4.3 If N/PN/NI to 4.1: Did the outcome assessors know which intervention measures the subjects have received? | NA/Y/PY/PN/N/NI | Y | Y | Y | Y | N | Y | N | Y | N | Y |
|  | 4.4 If Y/PY/NI to 4.3: Is it possible for the type of intervention received to affect the outcome measurement? | NA/Y/PY/PN/N/NI | PN | PN | PN | PN |  | PN |  | PN |  | PN |
|  | 4.5 If Y/PY/NI to 4.4: Did knowing the intervention measures affect the measurement of outcome variables? | NA/Y/PY/PN/N/NI |  |  |  |  |  |  |  |  |  |  |
|  |  |  | **Low** | **Low** | **Low** | **Low** | **Low** | **Low** | **Low** | **Low** | **Low** | **Low** |
| Bias in  selection of  the reported  result | 5.1 Whether the data analysis of the results is consistent with the pre-determined analysis plan made before unblinding the outcome data? | NA/Y/PY/PN/N/NI | Y | Y | Y | Y | Y | Y | Y | Y | Y | Y |
|  | 5.2 Multiple outcome measurements (e.g. scales, definitions, time points) within the outcome domain? | NA/Y/PY/PN/N/NI | N | Y | N | Y | N | N | N | N | N | N |
|  | 5.3 Multiple analyses of the data? | NA/Y/PY/PN/N/NI | N | N | N | N | N | N | N | N | N | N |
|  |  |  | **Low** | **High** | **Low** | **High** | **Low** | **Low** | **Low** | **Low** | **Low** | **Low** |
| **Overall bias** |  |  | **Low** | **High** | **Some concern** | **High** | **Low** | **Some concern** | **Low** | **Low** | **Low** | **Low** |
| Note: Y(Yes); PY(Probably Yes); N(No); PN(Probably No); NI(No Information); NA(Not Applicable). | | | | | | | | | | | | |

**Appendix 2.** Bias analysis results using version 2 of the Cochrane Risk-of-Bias tool for (RoB 2) for RCTs, Continued

|  |  | **Question** | **Option** | **Khamboon et al.**  **2021** | **Wu 2022** | **Si et al.**  **2021** | **Mi et al.**  **2018** | **Li et al.**  **2017** | **Xu et al.**  **2017** | **Wang et al.**  **2020** |
| --- | --- | --- | --- | --- | --- | --- | --- | --- | --- | --- |
| 1. Bias due to confounding |  | 1.1 Were there potential confounding factors that may affect the intervention effect in the study? |  | N | N | N | N | N | N | N |
|  |  | 1.2 Was the outcome analysis based on the follow-up time periods that were split due to intervention conversion among the participants? | N/PN：respond  1.4-1.6  Y/PY：respond 1.3 |  |  |  |  |  |  |  |
|  |  | 1.3 Was it possible that the interruption or conversion of the intervention is related to the prognostic factors of the outcome? | N/PN：respond 1.4-1.6  Y/PY：respond 1.7-1.8 |  |  |  |  |  |  |  |
|  | Confounding only exists at baseline. | 1.4 Did the authors use appropriate analytical methods to control for all important confounding factors? | Y/PY：respond 1.5 |  |  |  |  |  |  |  |
|  |  | 1.5 Did the authors measure variables that are effective and reliable in controlling confounding variables in the study? |  |  |  |  |  |  |  |  |
|  |  | 1.6 Did the authors control for any post-intervention variables that may have been affected by the intervention? |  |  |  |  |  |  |  |  |
|  | Baseline confounding and time-varying confounding coexist. | 1.7 Did the authors use appropriate analytical methods to control for all important confounding variables and time-varying confounding? | Y/PY：respond 1.8 |  |  |  |  |  |  |  |
|  |  | 1.8 Did the authors effectively and reliably control for confounding variables by measuring variables that are available in the study? |  |  |  |  |  |  |  |  |
|  |  |  |  | **Low** | **Low** | **Low** | **Low** | **Low** | **Low** | **Low** |
| 1. Bias in selection of participants into the study |  | 2.1 Were the study participants selected for inclusion in the study or analysis based on individual characteristics observed after the intervention began? | N/PN: respond 2.4  Y/PY: respond 2.2 | N | N | N | N | N | N | N |
|  |  | 2.2 Were the intervention-related variables that influence the selection of participants potentially related to the intervention itself? | Y/PY: respond 2.3 |  |  |  |  |  |  |  |
|  |  | 2.3 Did the intervention-related variables that affect selection also potentially influenced by the occurrence or cause of the outcomes? | Y/PY: respond 2.5 |  |  |  |  |  |  |  |
|  |  | 2.4 Were most of the follow-up times for study participants consistent with the start time of the intervention? | N/PN: respond 2.5 | Y | Y | Y | Y | Y | Y | Y |
|  |  | 2.5 Could the correction techniques used correct for selection bias? |  |  |  |  |  |  |  |  |
|  |  |  |  | **Low** | **Low** | **Low** | **Low** | **Low** | **Low** | **Low** |
| 3. Bias in classification of interventions |  | 3.1 Was the intervention group clearly defined? |  | Y | Y | Y | Y | Y | Y | Y |
|  |  | 3.2 Was information on the definition of the intervention group recorded at the start of the intervention? |  | Y | Y | Y | Y | Y | Y | Y |
|  |  | 3.3 Did the understanding of the outcome or outcome risk affect the classification of the intervention? |  | N | N | N | N | N | N | N |
|  |  |  |  | **Low** | **Low** | **Low** | **Low** | **Low** | **Low** | **Low** |
| 4. Bias due to deviations from intended interventions | If the research objective is to evaluate the results of intention-to-treat analysis, refer to section 4.1; if the research objective is to evaluate compliance and analytical results, refer to section 4.3. | 4.1 Were there any deviations from expected practices in the intention of the intervention beyond routine clinical practice? | Y/PY: respond 4.2 | N | N | N | N | N | N | N |
|  |  | 4.2 Were these deviations from intention interventions unevenly distributed among groups and may have affected the outcomes? |  |  |  |  |  |  |  |  |
|  |  | 4.3 Were the important accompanying interventions balanced among the intervention groups? | N/PN: respond 4.6 | Y | Y | Y | Y | Y | Y | Y |
|  |  | 4.4 Were the interventions successfully implemented for most of the participants? | N/PN: respond 4.6 | Y | Y | Y | Y | Y | Y | Y |
|  |  | 4.5 Did the study participants complied with the assigned intervention? | N/PN: respond 4.6 | Y | Y | Y | Y | Y | Y | Y |
|  |  | 4.6 Was appropriate analysis used in the study to assess the adherence effect of the intervention? |  |  |  |  |  |  |  |  |
|  |  |  |  | **Low** | **Low** | **Low** | **Low** | **Low** | **Low** | **Low** |
| 5. Bias due to missing data |  | 5.1 Was it possible to obtain outcome data for all or almost all participants in the study? | N/PN; respond 5.4-5.5 | Y | N | N | Y | Y | Y | Y |
|  |  | 5.2 Were participants excluded due to missing data on intervention status? | Y/PY: respond 5.4-5.5 | N | N | Y | N | N | N | N |
|  |  | 5.3 Were participants excluded due to missing data on other variables required for analysis? | Y/PY: respond 5.4-5.5 | N | N | N | N | N | N | N |
|  |  | 5.4 Was the proportion of missing data and the reasons for missing data similar between groups? |  |  | NI | N |  |  |  |  |
|  |  | 5.5 Was there evidence that the results remain robust after missing data? |  |  | N | N |  |  |  |  |
|  |  |  |  | **Low** | **High** | **High** | **Low** | **Low** | **Low** | **Low** |
| 6. Bias in measurement of outcomes |  | 6.1 Did knowledge of the intervention affect the measurement of the outcome? |  | NI | NI | NI | NI | NI | NI | NI |
|  |  | 6.2 Did outcome assessors know the interventions received by the participants? |  | N | Y | Y | Y | NI | Y | Y |
|  |  | 6.3 Were outcome assessment methods comparable between intervention groups? |  | Y | Y | Y | Y | Y | Y | Y |
|  |  | 6.4 Was there any systematic error in outcome measurement related to the intervention received? |  | N | NI | NI | NI | N | NI | NI |
|  |  |  |  | **Low** | **High** | **High** | **High** | **Moderate** | **High** | **High** |
| 7. Bias in selection of the reported result |  | 7.1 Was there any selective reporting of multiple outcome measurements within a specific outcome domain? |  | N | N | N | N | N | N | N |
|  |  | 7.2 Was there any selective reporting of effect sizes for multiple analyses of intervention-outcome relationships? |  | N | N | N | N | N | N | N |
|  |  | 7.3 Was there any selective reporting of different subgroups? |  | N | N | Y | Y | N | N | N |
|  |  |  |  | **Low** | **Low** | **High** | **High** | **Low** | **Low** | **Low** |
| **Overall bias** |  |  |  | **Low** | **High** | **High** | **High** | **Moderate** | **High** | **High** |
| Note: Y(Yes); PY (Probably Yes); N(No); PN (Probably No); NI (No Information); NA (Not Applicable). | | | | | | | | | | |

**Appendix 3.** Bias analysis results using the Risk-of-Bias in Non-randomized Studies of Interventions (ROBINS-I) tool for quasi-experimental trials


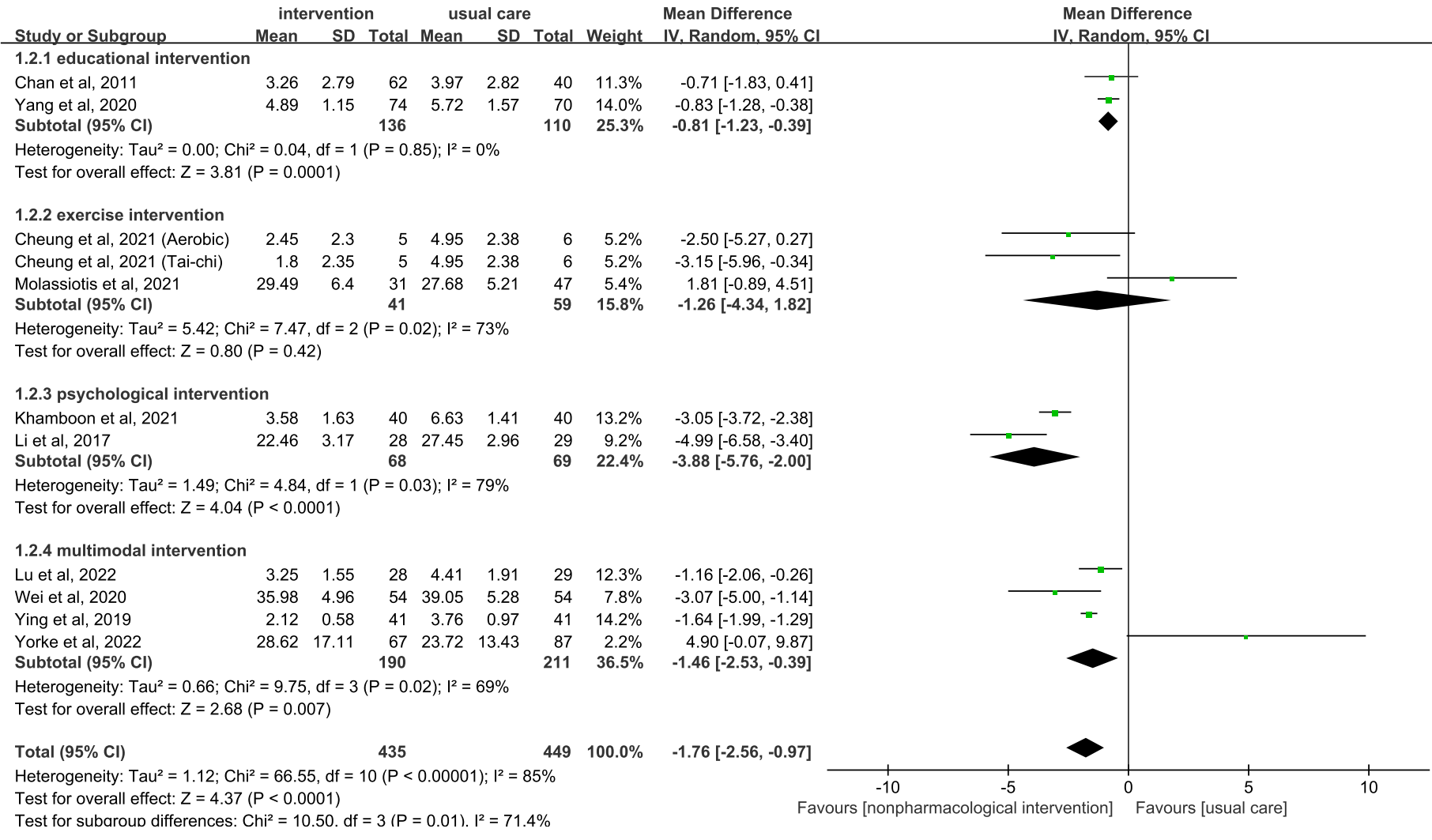


**Appendix 4.** Forest plot of non-pharmacological interventions on fatigue by intervention type subgroup


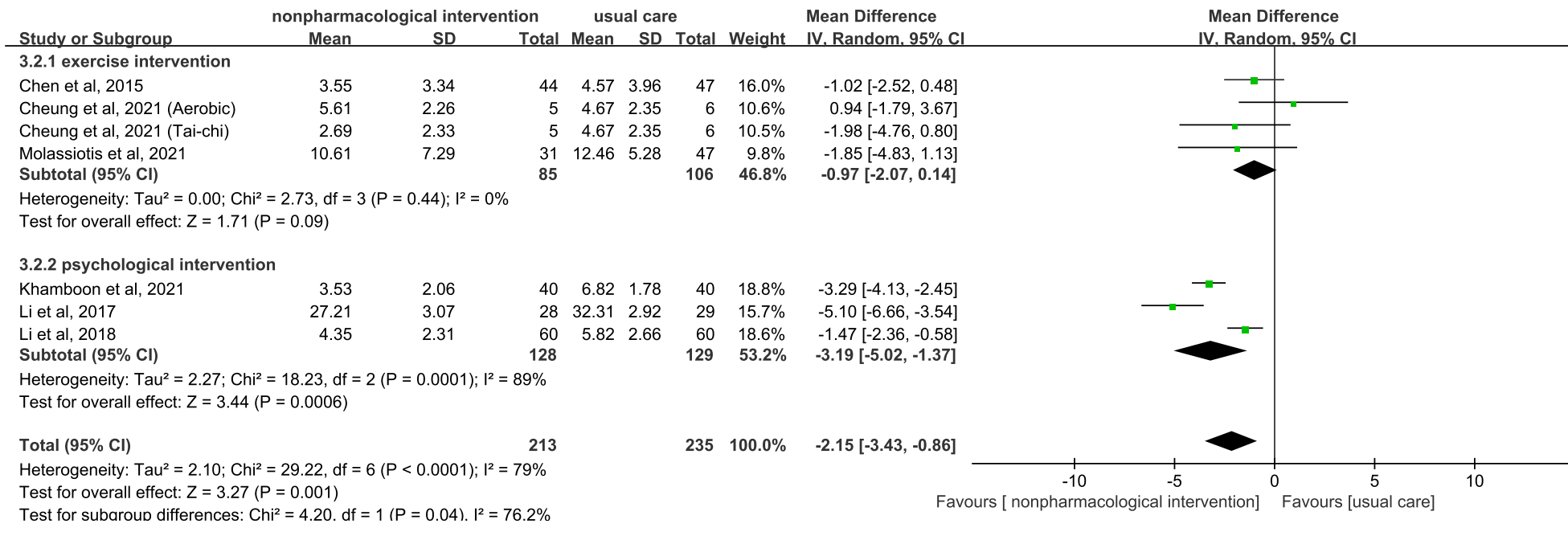


**Appendix 5.** Forest plot of non-pharmacological interventions on anxiety by intervention type subgroup


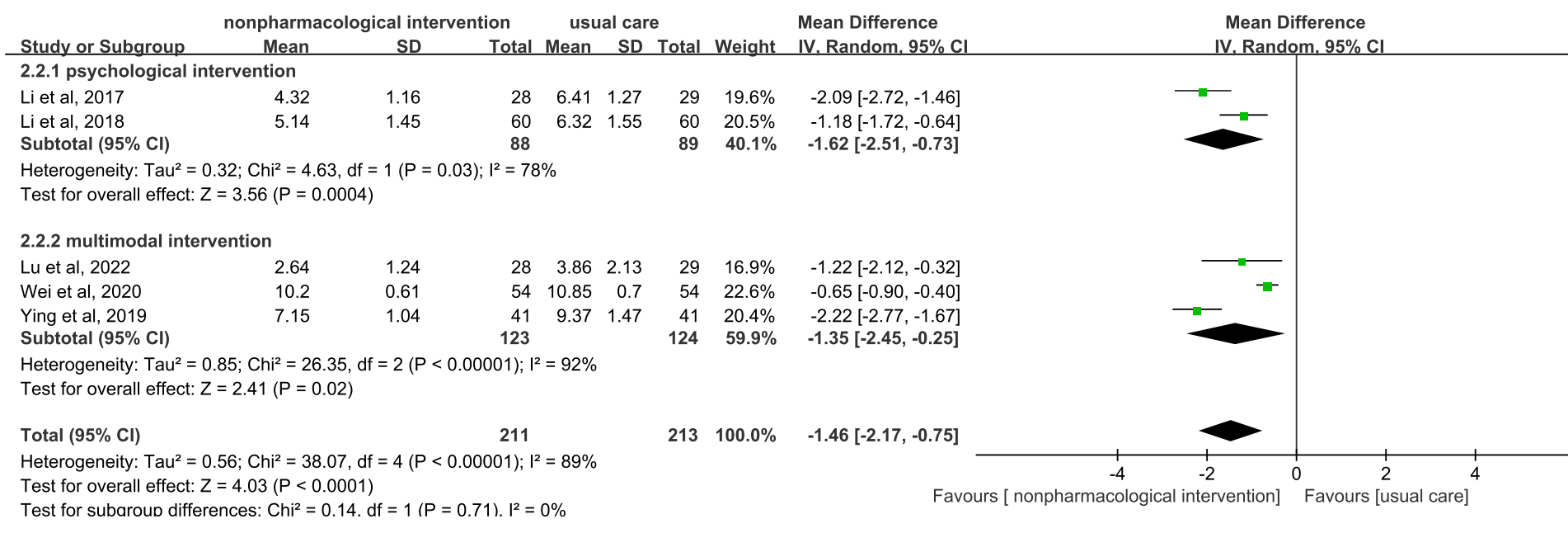


**Appendix 6.** Forest plot of non-pharmacological interventions on sleep disturbance by intervention type subgroup
